# Supplementary material for: Predictors of digital support services use by informal caregivers: a cross-sectional comparative survey
Source: BMJ Open. 2022 Apr 29;12(4):e059897. doi: 10.1136/bmjopen-2021-059897 (PMC9058775; doi:10.1136/bmjopen-2021-059897)
Supplement: Author's manuscript [file bmjopen-2021-059897.draft_revisions.pdf]

BMJ Open is committed to open peer review. As part of this commitment we make the peer review history of every article we publish publicly available.

When an article is published we post the peer reviewers' comments and the authors' responses online. We also post the versions of the paper that were used during peer review. These are the versions that the peer review comments apply to.

The versions of the paper that follow are the versions that were submitted during the peer review process. They are not the versions of record or the final published versions. They should not be cited or distributed as the published version of this manuscript.

BMJ Open is an open access journal and the full, final, typeset and author-corrected version of record of the manuscript is available on our site with no access controls, subscription charges or pay-per-view fees (http://bmjopen.bmj.com).

If you have any questions on BMJ Open's open peer review process please email info.bmjopen@bmj.com

BMJ Open

Predictors of Digital Support Services Use by Informal Caregivers: A Cross-Sectional Survey

Journal:	*BMJ Open*
Manuscript ID	bmjopen-2021-059897
Article Type:	Original research
Date Submitted by the Author:	08-Dec-2021
Complete List of Authors:	Hassan, Alhassan Yosri Ibrahim; National Institute of Health and Science on Aging, ; UNIVPM, Department of Economics and Social Sciences, Faculty of Economics "Giorgio Fuà"<br>Lamura, Giovanni ; National Institute of Health and Science on Aging,<br>Hagedoorn, Mariët; UMCG, University Medical Center Groningen
Keywords:	Health informatics < BIOTECHNOLOGY & BIOINFORMATICS, HEALTH ECONOMICS, Telemedicine < BIOTECHNOLOGY & BIOINFORMATICS, GERIATRIC MEDICINE, Quality in health care < HEALTH SERVICES ADMINISTRATION & MANAGEMENT, HEALTH SERVICES ADMINISTRATION & MANAGEMENT

SCHOLARONE™
Manuscripts

I, the Submitting Author has the right to grant and does grant on behalf of all authors of the Work (as defined in the below author licence), an exclusive licence and/or a non-exclusive licence for contributions from authors who are: i) UK Crown employees; ii) where BMJ has agreed a CC-BY licence shall apply, and/or iii) in accordance with the terms applicable for US Federal Government officers or employees acting as part of their official duties; on a worldwide, perpetual, irrevocable, royalty-free basis to BMJ Publishing Group Ltd ("BMJ") its licensees and where the relevant Journal is co-owned by BMJ to the co-owners of the Journal, to publish the Work in this journal and any other BMJ products and to exploit all rights, as set out in our licence.

The Submitting Author accepts and understands that any supply made under these terms is made by BMJ to the Submitting Author unless you are acting as an employee on behalf of your employer or a postgraduate student of an affiliated institution which is paying any applicable article publishing charge ("APC") for Open Access articles. Where the Submitting Author wishes to make the Work available on an Open Access basis (and intends to pay the relevant APC), the terms of reuse of such Open Access shall be governed by a Creative Commons licence – details of these licences and which Creative Commons licence will apply to this Work are set out in our licence referred to above.

Other than as permitted in any relevant BMJ Author's Self Archiving Policies, I confirm this Work has not been accepted for publication elsewhere, is not being considered for publication elsewhere and does not duplicate material already published. I confirm all authors consent to publication of this Work and authorise the granting of this licence.

For peer review only

Predictors of Digital Support Services Use by Informal Caregivers: A Cross-Sectional Survey

Alhassan Yosri Ibrahim Hassan; National Institute of Health and Science on Aging, Ancona, Italy; UNIVPM, Department of Economics and Social Sciences, Faculty of Economics "Giorgio Fuà", Ancona, Italy
Giovanni Lamura ; National Institute of Health and Science on Aging, Ancona, Italy
Mariët Hagedoorn,; University of Groningen, University Medical Center Groningen, Groningen, The Netherlands
Corresponding author:
Alhassan Yosri Ibrahim Hassan, BEng, MSc, MPH
National Institute of Health and Science on Aging
Via S. Margherita, 5 Ancona, Italy
Phone: +39 3884562527
Email: a.hassan@inrca.it; hassanyousri@hotmail.com
Keywords: informal caregivers; digital health; eHealth; health economics; home care
Word count: 3284

Abstract

Objectives Digital support services may provide informal caregivers with remote access to information and training about care issues. However, there is limited specific data on how factors such as demographics, socioeconomic resources and the caregiving context may influence caregivers' use of digital support services. The aim of this study is to identify associations between informal caregiver's characteristics and the use of the Internet to access digital support services in two countries: Italy and Sweden.

Setting and Participants A sample of 663 respondents participated in a cross-sectional survey by completing the online questionnaire. Respondents were recruited by the Italian National Institute of Health and Science on Ageing and the Swedish Family Care Competence Centre.

Primary and secondary outcome measures Logistic regression analyses were performed to assess predictors of caregivers' frequent use of the Internet to access digital support services.

Results Educational attainment (odds ratio [OR] 3.649, 95%CI: 1.424-9.350, p=0.007), hours per week spent caring (odds ratio [OR] 2.928, 95%CI: 1.481-5.791, p=0.002), total household income (odds ratio [OR] 0.378, 95%CI: 0.149-0.957, p=0.040), care recipient relationship to the caregiver (odds ratio [OR] 2.895, 95%CI: 1.037-8.083, p=0.042) and gender of care recipient (odds ratio [OR] 0.575, 95%CI: 0.356-0.928, p=0.023) were significant predictors in the multivariate analysis for the Italian caregivers group. Hours per week spent caring (odds ratio [OR] 2.401, 95%CI: 1.105-5.218, p=0.027) and age of care recipient (odds ratio [OR] 2.237,

For peer review only - http://bmjopen.bmj.com/site/about/guidelines.xhtml

95%CI: 1.150-4.352, p=0.018) were significant predictors in the multivariate analysis for the Swedish caregivers group.
Conclusions Digital support services could be important tools to empower informal caregivers. When it comes to policy and practice in relation to caregivers, similarly to other broad vulnerable groups, there is no 'one size fits all' approach, and it is therefore important to consider the specific characteristics and needs of both caregivers and care recipients.

Strengths and limitations of this study

- This study is an international comparative study investigating the important factors associated with the use of digital support services among informal caregivers
- Multivariate logistic regression analyses enabled the effect of confounding factors to be controlled for and predictors of use of digital support services among informal caregivers to be identified
- We provide evidence that there is scope for some categories of caregivers to be better supported with digital support services
- Given the cross-sectional design of our study, causal relationships cannot be established
- The survey was conducted using the internet, and thus our findings may not be generalizable to individuals who do not use the internet

For peer review only

Introduction

Informal caregivers are individuals who provide care to ill, frail or disabled relatives, friends or others, without being trained or paid, in contrast to formal caregivers who offer professional services [1]. In Europe, 80% of all care is provided by informal caregivers who are often females, either providing care to a spouse, parent or parent-in-law, and a large share is provided by individuals who are older than standard retirement age [2-4]. Estimates on the economic value of unpaid informal care in the European Union (EU) Member States range from 50% to 90% of the overall costs of formal long-term care provision [4]. The available estimates of the number of informal caregivers ranges from 10% up to 25% of the total population in Europe [5]. The number of informal caregivers over 18 years of age who provide more than 20 hours per week of informal care to older adults and relatives with disability is estimated to be more than 70 million [5]. Informal caregivers provide the bulk of long-term care, including via contributions to both activities of daily living (personal care, feeding, dressing and grooming, emotional and social support, etc.) as well as instrumental activities of daily living (transportation, care coordination, etc.)

Caregiving may prove challenging and stressful for many informal caregivers. Caregivers often experience high levels of need for information and services. Available literature points to the importance of novel technology solutions as a promising approach for empowering and supporting informal caregivers [6-8]. Digital support services for informal caregivers are services provided by any private or public organization that address caregivers and/or care recipients' needs through technological devices that are integrated or not into a wider intervention program [9]. Digital support services may provide informal caregivers with remote access to information and training about care and caring-related issues through websites, mobile applications and online training materials [10]. These solutions may contribute to a more positive caregiving experience and may help to strengthen informal caregivers' sense of social inclusion and belonging [11]. Digital support services also have macro-level benefits as these solutions may help in the integration of informal and formal care through better care coordination and a reduction in unnecessary hospitalizations and lengths of stay [8-12]. Consequently, the deployment of these solutions may generate savings and contribute to the sustainability of care systems [8-12].

Considering the substantial information needs experienced by informal caregivers, the increased availability of digital support services for caregivers as well as the potential they offer, further understanding of caregivers' use of the Internet to access digital support services is needed [13,14], in order to determine whether factors such as demographics, socioeconomic resources and the caregiving context may influence caregivers' use of digital support services [15]. Previous literature on Internet use for health information seeking showed that young age, good health status and higher education are associated with a more frequent use [16-28]. Prior

studies also found that females were likely to seek health information on the Internet more frequently than males [16-28]. In the literature, very few studies exclusively focus on caregivers' use of the Internet to access support services. While informal caregivers have been identified as a population group which could benefit from the provision of digital support services, there is limited specific data on how factors such as demographics, socioeconomic resources and the caregiving context may influence caregivers' use of the Internet to access digital support services. Mapping the sociodemographic and socioeconomic profiles of informal caregivers who do use and those who not use digital support services could help improve the quality of these services available to them. The aim of this study is therefore to identify associations between informal caregiver's characteristics and the use of the Internet to access digital support services in two countries: Italy and Sweden.

Italy and Sweden represent two European extremes with respect to several dimensions. These include: familistic/universalistic orientation of care system (Italy: family-based, Sweden: universal); the level of overall digital skills (low in Italy: 42%, high in Sweden: 72%); and that of Internet use for health information-seeking (low in Italy: 35%, high in Sweden: 62%) [29-32]. The two countries share however also some similarities. Both Italy and Sweden are high income countries and represent two of the oldest populations in Europe [33,34], also because they report an almost similar, very high life expectancy at birth, estimated at 83 and 82 years for Italy and Sweden, respectively [33,34]. Estimates on the prevalence of informal care in Italy ranges from 14% up to 26% of the country's population [35]. In Sweden, it is estimated that 18% of the 18+ population provides informal care on a regular basis, corresponding to over 1.3 million people overall [36]. Exploring the experiences of informal caregivers in accessing digital support services in these two countries could inform future reforms of the health care system, and boost caregivers' access to information, services and support via new technologies in accordance to their needs. Moreover, since health promotion and patient empowerment via digital technologies are also on the European agenda [37], exploring the commonalities and differences in informal caregivers' access to digital support services in these two countries, could contribute to provide recommendations useful for implementing the EU agenda on the transformation of the digital health and care agenda, while responding to caregivers' needs in each country.

For peer review only

Methods

Study Design

This online survey study used a cross-sectional design to identify associations between informal caregiver's characteristics and the use of Internet to access digital support services in two countries: Italy and Sweden. The data presented here, aimed at evaluating technology based support services for informal caregivers, were collected through the support of a partnership of different stakeholders belonging to the Eurocarers' network (European Association Working for Carers). They represent national level caregiver organizations in mostly EU Member States as well as research centers working on these topics, such as the Centre for Socio-Economic Research on Ageing of INRCA IRCCS (Italy's National Institute of Health and Science on Ageing), the Swedish Family Care Competence Centre, the University Medical Center Groningen (Netherlands), and the Department of Economics and Social Sciences of Marche Polytechnic University (Italy).

Survey Administration

The sample was identified from the registries of the Italian National Institute of Health and Science on Ageing and the Swedish Family Care Competence Centre. The online survey link was disseminated from November 2020 till April 2021 through the different communication channels of the Italian National Institute of Health and Science on Ageing and the Swedish Family Care Competence Centre. Study participants were included provided they were:

- informal caregivers of dependent adult individuals living at home;
- 18 years old and above;
- and either resident in Italy and able to understand Italian (for participants answering the Italian version of the questionnaire), or resident in Sweden and able to understand Swedish (for participants answering the Swedish version of the questionnaire).

Exclusion criteria were as follows:

- informal caregivers of pediatric patients;
- professional or paid caregivers.

The study sample included respondents who classified themselves as informal caregivers based on the survey question: "Do you provide unpaid care at home to an adult relative, neighbor or friend to help them take care of themselves?". Participants were asked to answer this question with "yes" or "no," and if they answered "yes," then they were asked to continue with the questionnaire. A unique identification number was provided to each participant and stored together with the survey results, in order to eliminate duplicate entries. The participants were given the option to save their responses and return to complete the survey, or they could edit or clear the replies and initiate the survey another time. Data was recorded in the system using a password-protected data extraction form.

For peer review only

Variables and Measurement

Guided by Wilson's model of information-seeking behavior [38], the previous survey on services for supporting family carers of older dependent people in Europe "EUROFAMCARE" [39], and empirical evidence in the literature [16-28], this study included the following sets of independent variables: caregiver's demographics; caregiver's socioeconomic resources; and caregiving context. The dependent variable in this study is informal caregivers' frequent use of the Internet to access digital support services. In the survey, caregivers were asked to report how frequently they were using the Internet to access digital support services. Those using the Internet at least several times per month to access digital support services were classified as "frequent users", while those accessing it less often were classified as "infrequent users". Three demographic measures were included: caregiver's age, caregiver's gender and caregiver's health status. Ages were measured in chronological years and grouped into three categories: 18 to 39, 40 to 59, and 60 or older. Gender was measured nominally and was grouped into male and female. Caregiver's health status was grouped into poor, fair and good. Measures of social and economic circumstances were the caregiver's educational attainment and their total household income. Educational attainment was grouped into primary, secondary, bachelor's degree and higher than bachelor's degree. Income was assessed by asking the caregiver about their "monthly household net income from all sources". In order to distribute the income by different income groups and enhance the cross-national comparability of results between the two countries involved in this study, Italy and Sweden, we referred to the official figures of the national median equivalized disposable annual income from the European Commission's European statistical system "Eurostat" [32]. We used these official figures in classifying the participants into three groups of household net income in each of these two countries:

- lower income group: income is less than below 50% of the national median equivalized disposable annual income. This is equivalent to an income lower than 5802 Euro in the case of Italy and an income lower than 9356 Euro in the case of Sweden;
- middle income group: income is between below 50 % of the national median equivalized disposable annual income and above 60 % of the national median equivalized disposable annual income. This is equivalent to an income between 5802 Euro and 19658 Euro in the case of Italy and an income between 9356 Euro and 26826 Euro in the case of Sweden;
- upper income group: income is higher than 19658 Euro in the case of Italy and higher than 26826 Euro in Sweden.

Caregiving context was assessed using the following variables: reported number of weekly hours of care provided to the care recipient; reported number of years spent providing care; age and gender of the care recipient; relationship between the care recipient and the caregiver; and the level of dependency of the care recipient. Responses concerning the average number of weekly hours of caregiving have been

For peer review only

grouped into four categories: 1) 10 hours or less, 2) 11 to 20 hours, 3) 21 to 40 hours and 4) more than 40 hours. Care duration was measured on the basis of the caregiver's reported length of care provision to the care recipient (in number of years), and respondents were classified into two groups: those caring for two years or less; and those caring for a longer time. The age of the care recipient was reported according to two groups: 60 years or less and more than 60 years. The gender of care recipients was grouped into male and female. Caregivers were requested to provide information about the person whom they care for, in order to assess the relationship with the care recipient (e.g. parents / parents-in-law, spouse/partner, friend/neighbor, child or other relative. The level of dependency of the care recipient on the caregiver was clustered in 2 groups: high dependency and low dependency.

Data Analysis

The data analysis was conducted in three stages. It began with univariate analyses including percentages to describe the characteristics of this sample of caregivers. At the second stage, the relationship between the outcome variable and the independent variables was examined using Pearson's chi-squared test with Yates' continuity correction. Differences between groups were considered significant at the 5% level ($p \leq 0.05$). Contingency tables have been assessed, before proceeding to logistic regression, to ensure there were no cells with expected frequencies of fewer than 5 to prevent biased estimates [40]. At the last stage, logistic regression analysis was used to establish the ability of each variable to predict caregivers' frequent use of the Internet to access digital support services while controlling the effects of other variables. Variables identified as statistically significant in the bivariate analysis were entered into logistic regression analysis for each measure of use of the Internet to access digital support services.

The logistic regression analyses produced odds ratios with 95% confidence intervals to identify predictors of each measure. Results are reported in odds ratios, which can be interpreted as the ratio of the probability that caregivers with a particular characteristic (e.g., male gender) will use the Internet frequently to access digital support services, over the probability they will use the Internet frequently to access digital support services, had they not this characteristic. Odds ratios that are higher than 1 indicate a positive association between a given variable and using the Internet frequently to access digital support services, while an odds ratio lower than 1 indicates a negative association. Statistical analyses were performed using SPSS software version 28.0 (IBM, Armonk, NY, USA).

Research Ethics Approval

Permission to conduct the study was granted by the committee of Marche Polytechnic University and was approved by the executive board on November 2, 2020 (1026353). Informal caregivers expressing interest in participating in the study were informed about the aim of the study, the expected time to complete the questionnaire, and that data would be stored by the Centre for Socio-Economic

For peer review only

Research on Ageing of the Italian National Institute of Health and Science on Ageing. Informed consent was obtained from all participants.

Patient and public involvement

Patients or the public were not involved in the design, or conduct, or reporting, or dissemination plans of our research.

For peer review only

Results

Sample Description

A total of 663 informal caregivers, 410 from Italy and 253 from Sweden, participated in the survey by completing the online questionnaire. Table 1 presents the overall characteristics of the sample. Females represented a majority of respondents in the Italian group. The median age of caregivers was 54 years while the median age of care recipients was 73 years. Most Italian participants were providing care to a parent (n = 163, 39.8%), to a female care recipient (n=223, 54.4%), spent more than 40 hours per week providing care (n=170, 41.5%) and had completed secondary school or lower (n = 254, 62%). Nearly half of the participants (n=196, 47.8%) had an annual household income of less than 19.658 Euro. The big majority of caregivers in the Italian sample (n=342, 83.4%) reported a fair or poor health status, provided care to a highly-dependent care recipient (n=329, 80.2%) and had been providing care for more than 2 years (n=287, 70%) (Table 1).
When compared to their Italian counterparts, both Swedish participants and their care recipients had a higher median age of 65 and 75 years respectively. Females made up a majority of participants in the Swedish sample. Most of the Swedish respondents reported providing care to a spouse/partner (n=97, 38.3%), a male care recipient (n=136, 53.8%), spent less than 10 hours per week providing care (n=112, 44.3%) and had completed a secondary school or lower (n = 149, 58.9%). Nearly half of the participants in the Swedish group (n=109, 43.1%) had annual household incomes less than 26.826 Euro. The majority of the caregivers in the Swedish sample (n=210, 83%) had a fair or poor health status, were caring for a highly dependent care recipient (n=139, 54.9%) and had been providing care for more than 2 years (n=142, 56.1%) (Table 1).

Table 1. Characteristics of the sample (total sample N =663)

Variables	Italian sample n = 410<br>n (%)	Swedish sample n = 253<br>n (%)
**Gender**		
Male	93 (22.7)	57 (22.5)
Female	317 (77.3)	196 (77.5)
**Age**		
Median	54	65
18-39	48 (11.7)	17 (6.7)
40-59	241 (58.8)	77 (30.4)
More than 60	121 (29.5)	159 (62.8)
**Health Status**		
Good	68 (16.6)	43 (17.0)
Fair	171 (41.7)	155 (61.3)
Poor	171 (41.7)	55 (21.7)

For peer review only

**Education**		
Primary	29 (7.1)	35 (13.8)
Secondary	225 (54.9)	114 (45.1)
Bachelor	114 (27.8)	61 (24.1)
Higher than bachelor's degree	42 (10.2)	43 (17.0)
**Income**		
Lower	39 (9.5)	17 (6.7)
Middle	157 (38.3)	92 (36.4)
Upper	214 (52.2)	144 (56.9)
**Care recipient relationship to caregiver**		
Parents (In law)	163 (39.8)	63 (24.9)
Spouse/Partner	64 (15.6)	97 (38.3)
Child	105 (25.6)	48 (19.0)
Friend/Neighbor	30 (7.3)	26 (10.3)
Other	48 (11.7)	19 (7.5)
**Gender of care recipient**		
Male	187 (45.6)	136 (53.8)
Female	223 (54.4)	117 (46.2)
**Age of care recipient**		
Median	73	75
60 or younger	160 (39.0)	73 (28.9)
More than 60	250 (61.0)	180 (71.1)
**Level of dependency of the care recipient**		
High dependency	329 (80.2)	139 (54.9)
Low dependency	81 (19.8)	114 (45.1)
**Hours spend caring each week**		
10 hours or less	115 (28.0)	112 (44.3)
11-20 hours	68 (16.6)	62 (24.5)
21-40 hours	57 (13.9)	30 (11.9)
More than 40 hours	170 (41.5)	49 (19.4)
**Number of years providing care**		
2 years or less	123 (30.0)	111 (43.9)
More than 2 years	287 (70.0)	142 (56.1)

For peer review only

Factors associated with caregivers' frequent use of the Internet to access digital support services

Table 2 shows the factors associated with caregivers' frequent use of the Internet to access digital support services in the bivariate analysis for each of the two countries of the study. In the Italian group, two-thirds of the respondents reported using the Internet at least several times per month to access digital support services. At the bivariate level, this was associated with two demographic variables, caregiver's age and health status, and two socio-economic measures, caregiver's educational attainment and total household income. Five measures of caregiving context – care recipient relationship to the caregiver, gender of care recipient, age of care recipient, hours per week spent caring and the level of dependency of the care recipient – were also linked to the frequent use of the Internet to access digital support services.

In the Swedish sample, 54.2 % of the participants reported using the Internet at least several times per month to access digital support services. In the bivariate analysis, caregiver's age was significantly associated with the frequent use of the Internet to access digital support services. Three measures of caregiving context were also linked with the frequent use of the Internet to access digital support services: care recipient relationship to the caregiver, age of care recipient and the number of hours spent caring each week. None of the measures of socio-economic resources was significantly associated with frequent use of the Internet to access digital support services in the Swedish sample.

For peer review only

Table 2. Factors associated with caregivers' frequent use of the Internet to access digital support services in the bivariate analysis

	Using the Internet at least several times per month to access digital support services			
	Italian sample n = 410		Swedish sample n = 253	
**Variables**	**n (%)**	**p[1]**	**n (%)**	**p**
**All respondents**	274 (66.8)		137 (54.2)	
**Gender**		0.123		0.344
Male	56 (60.2) [2]		34 (59.6)	
Female	218 (68.8)		103 (52.6)	
**Age**		0.010		0.035
18-39	23 (47.9)		6 (35.3)	
40-59	169 (70.1)		50 (64.9)	
More than 60	82 (67.8)		81 (50.9)	
**Health Status**		0.042		0.268
Good	37 (54.4)		35 (63.6)	
Fair	115 (67.3)		79 (51.0)	
Poor	122 (71.3)		23 (53.5)	
**Education**		0.008		0.901
Primary	12 (41.4)		20 (57.1)	
Secondary	161 (71.6)		60 (52.6)	
Bachelor's degree	76 (66.7)		32 (52.5)	
Higher than bachelor's degree	25 (59.5)		25 (58.1)	
**Income**		0.025		0.736
Lower	32 (82.1)		10 (58.8)	
Middle	110 (70.1)		47 (51.1)	
Upper	132 (61.7)		80 (55.6)	
**Care recipient relationship to caregiver**		< 0.001		0.014

Parents (In law)	95 (58.3)		23 (36.5)	
Spouse/Partner	49 (76.6)		55 (56.7)	
Child	88 (83.8)		33 (68.8)	
Friend/Neighbor	16 (53.3)		15 (57.7)	
Other	26 (54.2)		11 (57.9)	
**Gender of care recipient**		< 0.001		0.732
Male	141 (75.4)		75 (55.1)	
Female	133 (59.6)		62 (53.0)	
**Age of care recipient**		0.002		0.037
60 or younger	121 (75.6)		47 (64.4)	
More than 60	153 (61.2)		90 (50.0)	
**Level of dependency of the care recipient**		0.032		0.853
High dependency	228 (69.3)		76 (54.7)	
Low dependency	46 (56.8)		61 (53.5)	
**Hours spend caring each week**		< 0.001		0.022
10 hours or less	57 (49.6)		49 (43.8)	
11-20 hours	46 (67.6)		38 (61.3)	
21-40 hours	38 (66.7)		17 (56.7)	
More than 40 hours	133 (78.2)		33 (67.3)	
**Number of years providing care**		0.464		0.213
2 years or less	79 (64.2)		65 (58.6)	
More than 2 years	195 (67.9)		72 (50.7)	

Notes:
[1] Differences between groups were considered significant at the 5% level ($p \leq 0.05$)
[2] Male caregivers who are frequently using the Internet as a % of the total number of male caregivers in the sample.

For peer review only

Predictors of caregivers' frequent use of the Internet to access digital support services

Table 3 summarizes the results of the logistic regression analysis predicting caregivers' frequent use of the Internet to access digital support services. For the Italian sample, nine variables significantly associated with a frequent use of the Internet to access digital support services in the bivariate analysis were entered into logistic regression analysis to identify which were predictive: caregiver's age, health status, educational attainment, total household income, care recipient relationship to the caregiver, gender of care recipient, age of care recipient, number of weekly hours of care and the level of dependency of the care recipient. The multivariate analysis indicated that educational attainment, number of weekly hours of care, total household income, care recipient relationship to the caregiver and gender of care recipient remained significant predictors. The strongest predictor was the educational attainment of the caregivers. Informal caregivers who completed education equivalent to a Bachelor's degree level had 3.649 times the odds of using the Internet at least several times per month to access digital support services compared to those who completed a primary education (p=0.007, 95%CI: 1.424-9.350). Caregivers who spend more than 40 hours per week providing care were almost 3 times more likely to be frequent users of the Internet to access digital support services in comparison with those who spend 10 hours or less per week providing care. The odds of frequent use of the Internet to access digital support services were 2.646 times higher for caregivers belonging to the lower household income group compared to caregivers belonging to the upper household income group (p=0.040, 95%CI: 0.149-0.957). Regarding the relationship between the caregiver and care recipient, the caregivers of a child had 2.895 times the odds of using the Internet at least several times per month to access digital support services compared to those who provide care to another relative (p = 0.042, 95% CI: 1.037-8.083). The odds of frequently accessing digital support services were 1.739 times higher for caregivers who provide care to a male care recipient compared to those providing care to a female care recipient (p = 0.023, 95% CI: 0.356-0.928).
The logistic regression analysis to predict the frequent use of the Internet to access digital support services among Swedish participants consisted of the four statistically significant factors identified in the bivariate analysis: caregiver's age, care recipient relationship to the caregiver, age of care recipient and the number of weekly hours of care (Table 3). The number of weekly hours of care remained a significant predictor in the multivariate analysis for the Swedish sample and was the strongest predictor. Swedish respondents who spend more than 40 hours per week providing care were almost 2.5 times more likely to be frequent users of the Internet to access digital support services as opposed to those who dedicate 10 hours or less per week to care provision (p = 0.027, 95% CI: 1.105-5.218). The age of the caregiver also remained a significant predictor in the multivariate analysis. Caregivers in the age group 40-59 years were 2.237 times more likely to use the Internet at least several times per month to access digital support services in comparison with those of the age group 60+ years (p = 0.018, 95% CI: 1.150-4.352).

For peer review only

Table 3. Multivariate logistic regressions: caregivers' frequent use of the Internet to access digital support services

	**Using the Internet at least several times per month to access digital support services**					
	**Italian sample n = 410**			**Swedish sample n = 253**		
**Variables**	**p Value**	**OR**	**95% CIs**	**p Value**	**OR**	**95% CIs**
**Age (in years) (Ref.: 60+)**						
18-39	0.270	0.630	0.277-1.433	0.653	0.761	0.231-2.508
40-59	0.563	1.175	0.680-2.030	0.018	2.237	1.150-4.352
**Health Status (Ref.: Good)**				- [1]	-	-
Fair	0.703	1.105	0.661-1.850			
Poor	0.925	1.033	0.523-2.040			
**Education (Ref.: Primary)**				-	-	-
Secondary	0.008	3.236	1.358-7.711			
Bachelor	0.007	3.649	1.424-9.350			
Higher than bachelor's degree	0.077	2.624	0.901-7.647			
**Income (Ref.: Lower)**				-	-	-
Middle	0.170	0.514	0.198-1.331			
Upper	0.040	0.378	0.149-0.957			

**Care recipient relationship to caregiver (Ref.: Other)**						
Parents (In law)	0.554	0.797	0.376-1.688	0.086	0.370	0.119-1.150
Spouse/Partner	0.337	1.611	0.608-4.267	0.634	0.777	0.275-2.196
Child	0.042	2.895	1.037-8.083	0.911	1.075	0.302-3.828
Friend/Neighbor	0.673	0.806	0.297-2.192	0.885	1.095	0.320-3.744
**Gender of care recipient (Ref.: Male)**				-	-	-
Female	0.023	0.575	0.356-0.928			
**Age of care recipient (Ref.: 60 or younger)**						
More than 60	0.211	1.616	0.762-3.424	0.920	1.046	0.436-2.511
**Level of dependency of the care recipient (Ref.: High dependency)**				-	-	-
Low dependency	0.738	1.111	0.599-2.062			
**Hours spend caring each week (Ref.: 10 hours or less)**						
11-20 hours	0.021	2.241	1.127-4.459	0.085	1.822	0.921-3.602
21-40 hours	0.103	1.908	0.878-4.144	0.311	1.568	0.656-3.748
More than 40 hours	0.002	2.928	1.481-5.791	0.027	2.401	1.105-5.218

Notes:

Only variables significantly associated with using the Internet frequently to access digital support services in the bivariate analysis were entered into multivariate logistic regression analysis

For peer review only

Discussion

Principal findings

The purpose of this study was to identify important factors related to caregivers' use of the Internet to access digital support services in Italy and Sweden. The findings suggest that a number of demographic, socio-economic and caring circumstances are associated with the frequency of using the Internet to access digital support services among caregivers in both countries. Multivariate regression analyses enabled the effect of confounding factors to be controlled for and predictors of use to be identified. In consistency with literature [16-28], our findings indicate that caregiver's age, health status, caregiver's educational attainment, total household income, care recipient relationship to the caregiver, gender of care recipient, age of care recipient, hours per week spent caring and the level of dependency of the care recipient are all associated with use.
The study shows that more than half of the caregivers in both countries frequently use the Internet to access digital support services. While the use of the Internet for health information has been somewhat less common in Southern European countries, in our study the Italian and the Swedish groups report a similar use of the Internet to access digital support services. This may be related to the lower median age of the Italian sample compared to the Swedish one. Caregivers from Southern European countries with a family-based care system often lack support in terms of formal services and professional training from the government [41-43]. This shortcoming of support may increase their need for information and services. Digital support services may be an alternative support source that enables remote access to information and training about care and caring-related issues. Previous studies suggested that the use of the Internet for health information in Southern European countries is increasing, and that caregivers from this region are showing an increased interest in accessing new technologies aiming to support them [48-50].

In both countries, most of the caregivers who participated in the study were females, which is consistent with the results of previous works [25-28] and with the central role played by females in the provision of informal care [2-4]. In coherence with previous literature [39,41-48], the majority of Italian participants in our study provided care to a parent (in law) and spent more than 40 weekly hours of care, compared to their Swedish counterparts who provided care to spouse/partner and spent less than 10 hours per week providing care. Previous research showed that care for someone in one's own household is more common in Southern European countries than in Northern countries. In Southern countries, caregivers are more likely to live with their care recipients who often are parents / in-laws [39,41-48]. In Northern countries, in-household care is mostly spouse care, as it is rare for old persons to live with anyone else than their spouse. Consequently, caregivers from Southern European countries spend more hours in caregiving compared to caregivers from Northern countries.

As it is to be expected given previous research on using the Internet for general health information [19-24], the digital divide may negatively affect caregivers' use of

the Internet to access digital support services [51,52]. The socioeconomic status of users seems to be a significant factor that increases the digital divide in Southern European countries [53-55]. This was apparent in our study, showing that the divide was more significant in the case of the Italian group compared to the Swedish one. While none of the measures of socio-economic resources was significantly associated with a frequent use of the Internet to access digital support services in the Swedish group, the strongest predictor for the frequency of Internet use in the Italian group was the caregiver's educational attainment. Previous research has shown that better-educated caregivers are more likely to be engaged in more frequent online activities [25-28]. Income was also a predictor for the frequency of Internet use to access digital support services in the Italian group, with higher odds for caregivers belonging to the lower household income group. While literature suggests that general Internet users in higher-income households are more likely than others to go online frequently [56,57], previous studies on the Internet use for health-related activities suggest that lower-income households may be more likely than others to go online for support activities [16,58,59]. One possible explanation is that those with higher incomes may have other means of support, while those with lower incomes may turn to the Internet as an alternative source of assistance.

The literature shows that age is a factor associated with Internet use [16-28]. In the Swedish group of our study, age is a significant predictor of frequency of use. Age remained an important predictor of use when the effects of other demographics, socio-economic factors and caring circumstances had been controlled for. This suggests that the relationship between age and use among Swedish caregivers cannot be entirely explained by increased financial hardship in later life. Previous research suggests that use of the Internet for health information is relatively constant by age, until age 65 when it begins to decline [16].

Patterns of use among caregivers in both countries also seem to be shaped by the caring experience. The number of weekly hours of care was a significant predictor for the frequency of Internet use by participants in both countries. Evidence from literature suggests that high-intensity caregivers report higher levels of information and service needs [60]. Given the availability and convenience of online sources, high-intensity caregivers may turn to the Internet for digital support services.

Limitations

Some limitations concerning this study need to be considered. The risk of the typical sampling bias should be mentioned as higher income and more educated caregivers are more likely to participate in research studies involving modern technologies, which was the case in our study. Furthermore, the sample size, especially of the Swedish sample, prevented us from carrying out more sophisticated statistical analyses. Moreover, not all of those who provide informal caregiving and assistance to others identify themselves as informal caregivers; consequently, we may have failed to capture the experiences of these underrepresented groups. Although the most important variables identified from empirical evidence in the literature were

included in the models, residual external variables may still have influenced our results. Conclusions drawn from this study results must be tempered by the fact that respondents were already possessing minimal digital skills that would enable them to access online services. It is possible that those who are not interested or involved with technology or those with limited digital access are less likely to respond to online surveys; consequently, the data collected online might be skewed and the sample might be less representative for the population. These issues might have influenced our findings and underline the need to interpret the findings from this study and other studies on caregivers with some caution when generalizing the findings.

Recommendations and Implications

Our results indicate that digital support services may enable remote access to information and training about care and caring-related issues. In this context, looking for information and support services online may be considered an attempt to close some knowledge gap. With the rapid technology advancement and increased access to the Internet, more caregivers are expected to access these services [61]. This suggests that the interaction with informal caregiver by health care professionals and other parties with an interest in supporting them (e.g. caregiver advocacy organizations) is an integral part of the value chain that supports both communication and coordination of services. Hence, these parties should all be more engaged with developing digital support services targeted at informal caregivers, and carefully assess and identify their information and service needs. Consequently, better targeted information could be provided to caregivers through credible online sources. In this regard, an early assessment of caregivers' needs and digital skills demonstrates that large-scale actions aiming to equip informal caregivers with the digital skills they need to access digital support services are needed. This is key to enable informal caregivers to identify the available digital support services, and apply them to their own care situation. More research is therefore needed to examine the extent to which existing digital support services meet caregivers' information and service needs.

Addressing socio-economic inequalities is likely to be key to reduce the digital divide in caregivers' use of the Internet to access digital support services. As for the influence of age and education on the digital divide, health care professionals, service providers and social workers should pay particular attention to those caregivers who are older and less educated. Access to computers and Internet connections at public facilities, such as local libraries, community centers and senior centers should be provided with extra support to accommodate caregivers' information needs and overcome any barriers of use [15,19]. Moreover, seminars and campaigns on how to access digital support services could enhance caregivers' digital skills and experiences. Tailor-made campaigns and classes for older and less-educated adults are needed to help address any barriers related to their use of computers and new technology.

The finding that caregivers who indicate higher-intensity levels of caregiving are likely to engage in frequent Internet use to access digital support services may

For peer review only

suggest that the Internet could be used to reach out to these caregivers and meet their information and service needs. Online training materials, support groups, social networking systems for peer support and volunteer call networks could be used to reach out to caregivers [62]. Research is needed to further examine the effectiveness of digital support services in helping caregivers, if we are to improve these services and tailor them to the lives of those with substantial and unpredictable caring responsibilities.

Conclusions

The findings from this study can provide guidance and assistance for the deployment of digital support services for informal caregivers. Nevertheless, due to rapid technological innovation, especially in this sector, continuous research needs to be conducted and guidelines for developing digital support services should be made adaptable to ongoing and future changes. The care sector is undergoing a fast transformation and expansion also due to the direct and indirect effects of the COVID-19 pandemic. Health and social care delivery systems experience a technologically supported transition towards home care. New technologies are being developed for informal caregivers and these tools may well offer benefits to many of them. It is widely acknowledged that caregivers are a group with high levels of unmet needs when it comes to their access to information and other services. Digital support services could be important tools to empower and support informal caregivers. On the other hand, it also needs to be recognized that informal caregivers are a diverse population, living in a wide range of personal and social circumstances. When it comes to policy and practice in relation to caregivers, similarly to other broad vulnerable groups, there is no 'one size fits all' approach, and it is therefore important to consider the specific characteristics and needs of both caregivers and care recipients. Policy makers, health care professionals and all parties with an interest in supporting informal caregivers are encouraged to identify the outcomes that the latter regard as helpful, and to identify the interventions that can achieve such outcomes in consultation with them. This applies as much to the approach taken in relation to the development of digital support services as it does to other services. While digital support services have the potential to meet some of the needs of the caregivers, they cannot be seen as the only way to deliver information and support. These services represent only one of many instrument in a toolbox, and should therefore be tailored in a coordinated way with other existing services, such as respite care, access to training, and recognition of skills and work-life balance measures.

Contributorship statement

AH developed the research idea and wrote the manuscript. GL and MH were involved in the conceptualization of the project and provided critical evaluation and approval of the final submitted manuscript

Competing interests

For peer review only

None declared

Funding

This research was funded by the European Union's Horizon 2020 research and innovation program under the Marie Skłodowska-Curie grant agreement number 814072 for the 4-year innovative training network ENTWINE informal care.

Data sharing statement

Raw data cannot be made openly available as the participants provided an informed consent, which indicated that the data to be collected shall not be disclosed.

References

1- Roth DL, Fredman L, Haley WE. Informal caregiving and its impact on health: A reappraisal from population-based studies. The Gerontologist. 2015.
2- Ciccarelli N, Van Soest A. Informal caregiving, employment status and work hours of the 50+ population in Europe. De Economist. 2018.
3- Colombo, F, Llena-Nozal, A, Mercier, J and Tjadens, F, 2011, Help Wanted? Providing and paying for long-term care, OECD Health Policy Studies, OECD Publishing.
4-Eurocarers. Enabling carers to care: an EU strategy to support and empower informal carers. 2020. URL: https://eurocarers.org/download/5315/Eurocarers-Strategy_final.pdf
5- Zigante, Valentina. 2018. 'Informal Care in Europe: Exploring Formalisation, Availability and Quality.' Brussels: LSE Consulting for the European Commission, 2018.
6- Lamura G, Mnich E, Nolan M, Wojszel B, Krevers B, Mestheneos L, EUROFAMCARE Group. Family carers' experiences using support services in Europe: empirical evidence from the EUROFAMCARE study. Gerontologist 2008.
7-Lamura G, Di Rosa M, Papa R, Poli A, Barbabella F. Implications of the use of migrant care work and web-based services on family caregivers' health. Int J Care Caring 2019 Feb 28.
8-Carretero S, Stewart J, Centeno C, Barbabella F, Schmidt A, Lamontagne-Godwin F, et al. "Can technology-based services support long-term care challenges in home care?" Analysis of evidence from social innovation good practices across the EU: CARICT project summary report. 2012
9-Barbabella F, Schmidt A, Lamontagne-Godwin F, Rodrigues R, Ruppe G, Lamura G. Assessing the impact of ICT-based solutions for carers in europe: preliminary findings from the CARICT project.
10- Kluzer S, Redecker C, Mediavilla C. Long-term care challenges in an ageing society: The role of ICT and migrants results from a study on England, Germany, Italy and Spain (No. JRC58533). 2010.
11-Newman K, Wang AH, Wang AZ, Hanna D. The role of internet-based digital tools in reducing social isolation and addressing support needs among informal caregivers: a scoping review. BMC public health. 2019 Dec;19.
12-Carretero S, Stewart J, Centeno C. Information and communication technologies for informal carers and paid assistants: benefits from micro-, meso-, and macro-levels. Eur J Ageing 2015.

13-Read J, Blackburn C. Carers' perspectives on the internet: implications for social and health care service provision. British Journal of Social Work. 2005.
14- Washington, K., Meadows, S., Elliott, S., & Koopman, R. (2011). Information needs of informal caregivers of older adults with chronic health conditions. Patient Education and Counseling,
15- Hassan AY. Challenges and recommendations for the deployment of information and communication technology solutions for informal caregivers: scoping review. JMIR aging. 2020.
16- Bundorf MK, Wagner TH, Singer SJ, Baker LC. Who searches the internet for health information?. Health services research. 2006.
17- Basch EM, Thaler HT, Shi W, Yakren S, Schrag D. Use of information resources by patients with cancer and their companions. Cancer: Interdisciplinary International Journal of the American Cancer Society. 2004.
18-Shaffer KM, Chow PI, Cohn WF, Ingersoll KS, Ritterband LM. Informal caregivers' use of internet-based health resources: An analysis of the health information national trends survey. JMIR aging. 2018.
19-Brodie M, Flournoy RE, Altman DE, Blendon RJ, Benson JM, Rosenbaum MD. Health Information, The Internet, And The Digital Divide: Despite recent improvements, Americans' access to the Internet—and to the growing body of health information there—remains uneven. Health affairs. 2000.
20- Czaja SJ, Sharit J, Hernandez MA, Nair SN, Loewenstein D. Variability among older adults in Internet health information-seeking performance. Gerontechnology. 2010.
21- Baker L, Wagner TH, Singer S, Bundorf MK. Use of the Internet and e-mail for health care information: results from a national survey. Jama. 2003.
22-Ybarra M, Suman M. Reasons, assessments and actions taken: sex and age differences in uses of Internet health information. Health education research. 2008.
23- Bass SB, Ruzek SB, Gordon TF, Fleisher L, McKeown-Conn N, Moore D. Relationship of Internet health information use with patient behavior and self-efficacy: experiences of newly diagnosed cancer patients who contact the National Cancer Institute's Cancer Information Service. Journal of Health Communication. 2006.
24- Flynn KE, Smith MA, Freese J. When do older adults turn to the internet for health information? Findings from the Wisconsin Longitudinal Study. Journal of general internal medicine. 2006.
25- Li H. Informal caregivers' use of the internet for caregiving information. Social work in health care. 2015.
26- Meyer K, Gassoumis ZD, Kelly K, Benton D. What are the characteristics of caregivers logging in for support services?. Innovation in aging. 2019.
27- Buchanan RJ, Huang C, Crudden A. Use of the internet by informal caregivers assisting people with multiple sclerosis. Journal of technology in human services. 2012.
28- Blackburn C, Read J, Hughes N. Carers and the digital divide: factors affecting Internet use among carers in the UK. Health & Social Care in the Community. 2005.

For peer review only

29-Garcés J, Ródenas F, Hammar T. Converging methods to link social and health care systems and informal care—confronting Nordic and Mediterranean approaches. In Long-Term Care in Europe 2013.
30- Nies H, Leichsenring K, Mak S. The emerging identity of long-term care systems in Europe. InLong-Term Care in Europe 2013 . Palgrave Macmillan, London.
31- Schulmann K, Leichsenring K, Genta M, Grigaliūnienė Ž, Kucsera C, Matei A, Määttänen N, Naegele G, Paat G, Ahi SP, Principi A. Social support and long-term care in EU care regimes. Framework Conditions and Initiatives of Social Innovation in an Active Ageing Perspective. Overview Report. WP8 MoPAct Project. 2014.
32- European Commission's European statistical system "Eurostat": https://ec.europa.eu/eurostat/web/main/home
33-World Health Organization . Italy: WHO Statistical Profile. World Health Organization; Geneva, Switzerland: 2015.
34-World Health Organization . Sweden: WHO Statistical Profile. World Health Organization; Geneva, Switzerland: 2015.
35-Eurocarers. Country profiles: Italy. https://eurocarers.org/country-profiles/italy/
36-Eurocarers. Country profiles: Sweden. https://eurocarers.org/country-profiles/Swden/
37- Draguet V. EU4Health 2021–2027–a vision for a healthier European Union. Public Health–European Commission
38- Wilson TD. Models in information behaviour research. Journal of documentation. 1999 Aug 1.
39- Lamura G, Mnich E, Nolan M, Wojszel B, Krevers B, Mestheneos L, Döhner H. Family carers' experiences using support services in Europe: empirical evidence from the EUROFAMCARE study. The Gerontologist. 2008.
40- Haberman SJ. A warning on the use of chi-squared statistics with frequency tables with small expected cell counts. Journal of the American Statistical Association. 1988.
41-Ferre F, de Belvis AG, Valerio L, Longhi S, Lazzari A, Fattore G, et al. Italy: health system review. Health Syst Transit. 2014.
42- Sansoni J, Vellone E, Piras G. Anxiety and depression in community-dwelling, Italian Alzheimer's disease caregivers. Int J Nurs Pract. 2004.
43- Wulff J, Fänge AM, Lethin C, Chiatti C. Self-reported symptoms of depression and anxiety among informal caregivers of persons with dementia: a cross-sectional comparative study between Sweden and Italy. BMC Health Services Research. 2020.
44-Castiello M, del Barrio É, Castejon P, Tortosa M, Sundström G, Malmberg B, Johansson L. Family care for elders in Europe: Policies and practices.
45- Quattrini S, Melchiorre MG, Balducci C, Spazzafumo L, Lamura G. Services for Supporting Family Carers of Older Dependent People in Europe: Characteristics, Coverage and Usage. The National Survey Report for Italy. 2006.
46- Szinovacz ME, Davey A, editors. Caregiving contexts: Cultural, familial, and societal implications. Springer Publishing Company; 2007.
47- Glendinning C, Tjadens F, Arksey H, Morée M, Moran N, Nies H. Care provision within families and its socio-economic impact on care providers. Heslington, York: Social Policy Research Unit, University of York; 2009.

48- Eurocarers/IRCCS-INRCA (2021). Impact of the COVID-19 outbreak on informal carers across Europe – Final report. Brussels/Ancona.
49- Kummervold PE, Wynn R. Health information accessed on the internet: the development in 5 European countries. International Journal of Telemedicine and Applications. 2012.
50- Athanasopoulou C, Välimäki M, Koutra K, Löttyniemi E, Bertsias A, Basta M, Vgontzas AN, Lionis C. Internet use, eHealth literacy and attitudes toward computer/internet among people with schizophrenia spectrum disorders: a cross-sectional study in two distant European regions. BMC medical informatics and decision making. 2017.
51-Burton LC, Newsom JT, Schulz R, Hirsch CH, German PS. Preventive health behaviors among spousal caregivers. Preventive medicine. 1997.
52-Given B, Sherwood PR, Given CW. What knowledge and skills do caregivers need?. Journal of Social Work Education. 2008.
53- Gourova E, Antonova A. Bridging the digital divide in South-Eastern Europe. IV International Bulgarian-Greak Scientific Conference Computer Science. 2008.
54-Dutta S, Geiger T, Lanvin B. The global information technology report 2015. In World Economic Forum. 2015.
55- Polykalas SE. Assessing the evolution of the digital divide across European Union. In2014 International Conference on Web and Open Access to Learning (ICWOAL) 2014.
56- Bansil P, Keenan NL, Zlot AI, Gilliland JC. Peer reviewed: health-related information on the web: results from the HealthStyles Survey, 2002–2003. Preventing chronic disease. 2006.
57- Jansen J. Use of the internet in higher-income households. Washington, DC: Pew Research Center; 2010.
58- Atkinson N, Saperstein S, Pleis J. Using the internet for health-related activities: findings from a national probability sample. Journal of medical Internet research. 2009.
59-Lee SY, Hwang H, Hawkins R, Pingree S. Interplay of negative emotion and health self-efficacy on the use of health information and its outcomes. Communication Research. 2008.
60- Li H. Unmet service needs: a comparison between dementia and non-dementia caregivers. Home health care services quarterly. 2012.
61-Fox S, Brenner J. Family caregivers online. Washington, DC: Pew Internet & American Life Project. 2012.
62- White MH, Dorman SM. Online support for caregivers. Analysis of an Internet Alzheimer mail group. Computers in nursing. 2000.

For peer review only

STROBE Statement—Checklist of items that should be included in reports of ***cross-sectional studies***

	Item No	Recommendation	Page No
**Title and abstract**	1	(*a*) Indicate the study's design with a commonly used term in the title or the abstract	1
		(*b*) Provide in the abstract an informative and balanced summary of what was done and what was found	1-2
**Introduction**			
Background/rationale	2	Explain the scientific background and rationale for the investigation being reported	3-4
Objectives	3	State specific objectives, including any prespecified hypotheses	3-4
**Methods**			
Study design	4	Present key elements of study design early in the paper	5
Setting	5	Describe the setting, locations, and relevant dates, including periods of recruitment, exposure, follow-up, and data collection	5
Participants	6	(*a*) Give the eligibility criteria, and the sources and methods of selection of participants	5
Variables	7	Clearly define all outcomes, exposures, predictors, potential confounders, and effect modifiers. Give diagnostic criteria, if applicable	6
Data sources/ measurement	8*	For each variable of interest, give sources of data and details of methods of assessment (measurement). Describe comparability of assessment methods if there is more than one group	*6*
Bias	9	Describe any efforts to address potential sources of bias	6
Study size	10	Explain how the study size was arrived at	6
Quantitative variables	11	Explain how quantitative variables were handled in the analyses. If applicable, describe which groupings were chosen and why	6-7
Statistical methods	12	(*a*) Describe all statistical methods, including those used to control for confounding	7-8
		(*b*) Describe any methods used to examine subgroups and interactions	7-8
		(*c*) Explain how missing data were addressed	7-8
		(*d*) If applicable, describe analytical methods taking account of sampling strategy	7-8
		(*e*) Describe any sensitivity analyses	7-8
**Results**			
Participants	13*	(a) Report numbers of individuals at each stage of study—eg numbers potentially eligible, examined for eligibility, confirmed eligible, included in the study, completing follow-up, and analysed	9
		(b) Give reasons for non-participation at each stage	9
		(c) Consider use of a flow diagram	9
Descriptive data	14*	(a) Give characteristics of study participants (eg demographic, clinical, social) and information on exposures and potential confounders	10-11
		(b) Indicate number of participants with missing data for each variable of interest	10-11
Outcome data	15*	Report numbers of outcome events or summary measures	12-17

For peer review only

For peer review only - http://bmjopen.bmj.com/site/about/guidelines.xhtml

Main results	16	(*a*) Give unadjusted estimates and, if applicable, confounder-adjusted estimates and their precision (eg, 95% confidence interval). Make clear which confounders were adjusted for and why they were included	12-17
		(*b*) Report category boundaries when continuous variables were categorized	12-17
		(*c*) If relevant, consider translating estimates of relative risk into absolute risk for a meaningful time period	12-17
Other analyses	17	Report other analyses done—eg analyses of subgroups and interactions, and sensitivity analyses	12-17
**Discussion**			
Key results	18	Summarise key results with reference to study objectives	18-19
Limitations	19	Discuss limitations of the study, taking into account sources of potential bias or imprecision. Discuss both direction and magnitude of any potential bias	19-20
Interpretation	20	Give a cautious overall interpretation of results considering objectives, limitations, multiplicity of analyses, results from similar studies, and other relevant evidence	20-21
Generalisability	21	Discuss the generalisability (external validity) of the study results	20-21
**Other information**			
Funding	22	Give the source of funding and the role of the funders for the present study and, if applicable, for the original study on which the present article is based	22

*Give information separately for exposed and unexposed groups.

Note: An Explanation and Elaboration article discusses each checklist item and gives methodological background and published examples of transparent reporting. The STROBE checklist is best used in conjunction with this article (freely available on the Web sites of PLoS Medicine at http://www.plosmedicine.org/, Annals of Internal Medicine at http://www.annals.org/, and Epidemiology at http://www.epidem.com/). Information on the STROBE Initiative is available at www.strobe-statement.org.

For peer review only

For peer review only - http://bmjopen.bmj.com/site/about/guidelines.xhtml

BMJ Open

Predictors of Digital Support Services Use by Informal Caregivers: A Cross-Sectional Comparative Survey

Journal:	*BMJ Open*
Manuscript ID	bmjopen-2021-059897.R1
Article Type:	Original research
Date Submitted by the Author:	07-Mar-2022
Complete List of Authors:	Hassan, Alhassan Yosri Ibrahim; National Institute of Health and Science on Aging, ; UNIVPM, Department of Economics and Social Sciences, Faculty of Economics "Giorgio Fuà"<br>Lamura, Giovanni ; National Institute of Health and Science on Aging,<br>Hagedoorn, Mariët; UMCG, University Medical Center Groningen
**Primary Subject Heading**:	Health informatics
Secondary Subject Heading:	Global health, Health economics, Health informatics, Health policy, Health services research
Keywords:	Health informatics < BIOTECHNOLOGY & BIOINFORMATICS, HEALTH ECONOMICS, Telemedicine < BIOTECHNOLOGY & BIOINFORMATICS, GERIATRIC MEDICINE, Quality in health care < HEALTH SERVICES ADMINISTRATION & MANAGEMENT, HEALTH SERVICES ADMINISTRATION & MANAGEMENT

SCHOLARONE™
Manuscripts

I, the Submitting Author has the right to grant and does grant on behalf of all authors of the Work (as defined in the below author licence), an exclusive licence and/or a non-exclusive licence for contributions from authors who are: i) UK Crown employees; ii) where BMJ has agreed a CC-BY licence shall apply, and/or iii) in accordance with the terms applicable for US Federal Government officers or employees acting as part of their official duties; on a worldwide, perpetual, irrevocable, royalty-free basis to BMJ Publishing Group Ltd ("BMJ") its licensees and where the relevant Journal is co-owned by BMJ to the co-owners of the Journal, to publish the Work in this journal and any other BMJ products and to exploit all rights, as set out in our licence.

The Submitting Author accepts and understands that any supply made under these terms is made by BMJ to the Submitting Author unless you are acting as an employee on behalf of your employer or a postgraduate student of an affiliated institution which is paying any applicable article publishing charge ("APC") for Open Access articles. Where the Submitting Author wishes to make the Work available on an Open Access basis (and intends to pay the relevant APC), the terms of reuse of such Open Access shall be governed by a Creative Commons licence – details of these licences and which Creative Commons licence will apply to this Work are set out in our licence referred to above.

Other than as permitted in any relevant BMJ Author's Self Archiving Policies, I confirm this Work has not been accepted for publication elsewhere, is not being considered for publication elsewhere and does not duplicate material already published. I confirm all authors consent to publication of this Work and authorise the granting of this licence.

For peer review only

Predictors of Digital Support Services Use by Informal Caregivers: A Cross-Sectional Comparative Survey

Alhassan Yosri Ibrahim Hassan; 1)National Institute of Health and Science on Aging, Ancona, Italy; 2)Marche Polytechnic University, Department of Economics and Social Sciences, Faculty of Economics "Giorgio Fuà", Ancona, Italy
Giovanni Lamura ; National Institute of Health and Science on Aging, Ancona, Italy
Mariët Hagedoorn,; University of Groningen, University Medical Center Groningen, Groningen, The Netherlands
Corresponding author:
Alhassan Yosri Ibrahim Hassan, BEng, MSc, MPH
National Institute of Health and Science on Aging
Via S. Margherita, 5 Ancona, Italy
Phone: +39 3884562527
Email: a.hassan@inrca.it; hassanyousri@hotmail.com
Keywords: informal caregivers; digital health; eHealth; health economics; home care
Word count: 3284

Abstract

Objectives Digital support services may provide informal caregivers with remote access to information and training about care issues. However, there is limited specific data on how factors such as demographics, socioeconomic resources and the caregiving context may influence caregivers' use of digital support services. The aim of this study is to identify associations between informal caregiver's characteristics and the use of the Internet to access digital support services in two countries: Italy and Sweden.

Setting and Participants A sample of 663 respondents who have access to the Internet participated in a cross-sectional survey by completing the online questionnaire. Respondents were recruited by the Italian National Institute of Health and Science on Ageing and the Swedish Family Care Competence Centre.

Primary and secondary outcome measures Logistic regression analyses were performed to assess predictors of caregivers' frequent use of the Internet to access digital support services.

Results Educational attainment (odds ratio [OR] 3.649, 95%CI: 1.424-9.350, p=0.007), hours per week spent caring (odds ratio [OR] 2.928, 95%CI: 1.481-5.791, p=0.002), total household income (odds ratio [OR] 0.378, 95%CI: 0.149-0.957, p=0.040), care recipient relationship to the caregiver (odds ratio [OR] 2.895, 95%CI: 1.037-8.083, p=0.042) and gender of care recipient (odds ratio [OR] 0.575, 95%CI: 0.356-0.928, p=0.023) were significant predictors in the multivariate analysis for the Italian caregivers group. Hours per week spent caring (odds ratio [OR] 2.401, 95%CI: 1.105-5.218, p=0.027) and age of care recipient (odds ratio [OR] 2.237,

95%CI: 1.150-4.352, p=0.018) were significant predictors in the multivariate analysis for the Swedish caregivers group.
Conclusions Digital support services could be important tools to empower informal caregivers. When it comes to policy and practice in relation to caregivers, similarly to other broad vulnerable groups, there is no 'one size fits all' approach, and it is therefore important to consider the specific characteristics and needs of both caregivers and care recipients.

Strengths and limitations of this study

- This study is an international comparative study investigating the important factors associated with the use of digital support services among informal caregivers
- Multivariate logistic regression analyses enabled the effect of confounding factors to be controlled for and predictors of use of digital support services among informal caregivers to be identified
- Given the cross-sectional design of our study, causal relationships cannot be established
- The survey was conducted using the internet, and thus our findings may not be generalizable to individuals who do not use the internet

For peer review only

Introduction

Informal caregivers are individuals who provide care to ill, frail or disabled relatives, friends or others, without being trained or paid, in contrast to formal caregivers who offer professional services [1]. In Europe, 80% of all care is provided by informal caregivers who are often females, either providing care to a spouse, parent or parent-in-law, and a large share is provided by individuals who are older than standard retirement age [2-4]. Estimates on the economic value of unpaid informal care in the European Union (EU) Member States range from 50% to 90% of the overall costs of formal long-term care provision [4]. The available estimates of the number of informal caregivers ranges from 10% up to 25% of the total population in Europe [5]. The number of informal caregivers over 18 years of age who provide more than 20 hours per week of informal care to older adults and relatives with disability is estimated to be more than 70 million [5]. Informal caregivers provide the bulk of long-term care, including via contributions to both activities of daily living (personal care, feeding, dressing and grooming, emotional and social support, etc.) as well as instrumental activities of daily living (transportation, care coordination, etc.)

Caregiving may prove challenging and stressful for many informal caregivers. Caregivers often experience high levels of need for information and services. Available literature points to the importance of novel technology solutions as a promising approach for empowering and supporting informal caregivers [6-8]. Digital support services for informal caregivers are services provided by any private or public organization that address caregivers and/or care recipients' needs through technological devices that are integrated or not into a wider intervention program [9]. Digital support services may provide informal caregivers with remote access to information and training about care and caring-related issues through websites, mobile applications and online training materials [10]. These solutions may contribute to a more positive caregiving experience and may help to strengthen informal caregivers' sense of social inclusion and belonging [11]. Digital support services also have macro-level benefits as these solutions may help in the integration of informal and formal care through better care coordination and a reduction in unnecessary hospitalizations and lengths of stay [8-12]. Consequently, the deployment of these solutions may generate savings and contribute to the sustainability of care systems [8-12].

Considering the substantial information needs experienced by informal caregivers, the increased availability of digital support services for caregivers as well as the potential they offer, further understanding of caregivers' use of the Internet to access digital support services is needed [13,14], in order to determine whether factors such as demographics, socioeconomic resources and the caregiving context may influence caregivers' use of digital support services [15]. Previous literature on Internet use for health information seeking showed that young age, good health status and higher education are associated with a more frequent use [16-28]. Prior

studies also found that females were likely to seek health information on the Internet more frequently than males [16-28].

Italy and Sweden represent two European extremes with respect to several dimensions. These include: familistic/universalistic orientation of care system (Italy: family-based, Sweden: universal); the level of overall digital skills (low in Italy: 42%, high in Sweden: 72%); and that of Internet use for health information-seeking (low in Italy: 35%, high in Sweden: 62%) [29-32]. The two countries share however also some similarities. Both Italy and Sweden are high income countries and represent two of the oldest populations in Europe [33,34], also because they report an almost similar, very high life expectancy at birth, estimated at 83 and 82 years for Italy and Sweden, respectively [33,34]. Estimates on the prevalence of informal care in Italy ranges from 14% up to 26% of the country's population [35]. In Sweden, it is estimated that 18% of the 18+ population provides informal care on a regular basis, corresponding to over 1.3 million people overall [36].

In the literature, very few studies exclusively focus on caregivers' use of the Internet to access support services. While informal caregivers have been identified as a population group which could benefit from the provision of digital support services, there is limited specific data on how factors such as demographics, socioeconomic resources and the caregiving context may influence caregivers' use of the Internet to access digital support services. Mapping the sociodemographic and socioeconomic profiles of informal caregivers who do use and those who not use digital support services could help improve the quality of these services available to them. The aim of this study is therefore to identify associations between informal caregiver's characteristics and the use of the Internet to access digital support services in two countries: Italy and Sweden. Exploring the experiences of informal caregivers in accessing digital support services in these two countries could inform future reforms of the health care system, and boost caregivers' access to information, services and support via new technologies in accordance to their needs. Moreover, since health promotion and patient empowerment via digital technologies are also on the European agenda [37], exploring the commonalities and differences in informal caregivers' access to digital support services in these two countries, could contribute to provide recommendations useful for implementing the EU agenda on the transformation of the digital health and care agenda, while responding to caregivers' needs in each country.

Methods

Study Design

This online survey study used a cross-sectional design to identify associations between informal caregiver's characteristics and the use of Internet to access digital support services in two countries: Italy and Sweden. The data presented here, aimed at evaluating technology based support services for informal caregivers, were collected through the support of a partnership of different stakeholders belonging to the Eurocarers' network (European Association Working for Carers). They represent national level caregiver organizations in mostly EU Member States as well as research centers working on these topics, such as the Centre for Socio-Economic

Research on Ageing of INRCA IRCCS (Italy's National Institute of Health and Science on Ageing), the Swedish Family Care Competence Centreand the Department of Economics and Social Sciences of Marche Polytechnic University (Italy).

Survey Administration

The sample was identified from the registries of the Italian National Institute of Health and Science on Ageing and the Swedish Family Care Competence Centre. The online survey link was disseminated from November 2020 till April 2021 through the different communication channels, i.e., mailing lists and official websites, of the Italian National Institute of Health and Science on Ageing and the Swedish Family Care Competence Centre. Study participants were included provided they were:

- informal caregivers of dependent adult individuals living at home with access to the Internet;
- 18 years old and above;
- and either resident in Italy and able to understand Italian (for participants answering the Italian version of the questionnaire), or resident in Sweden and able to understand Swedish (for participants answering the Swedish version of the questionnaire).

Exclusion criteria were as follows:

- informal caregivers of pediatric patients;
- professional or paid caregivers.
- People with medical comorbidities that prevent them from completing the questionnaire (e.g., cognitive impairments)

The study sample included respondents who classified themselves as informal caregivers based on the survey question: "Do you provide unpaid care at home to an adult relative, neighbor or friend to help them take care of themselves?". Participants were asked to answer this question with "yes" or "no," and if they answered "yes," then they were asked to continue with the questionnaire. A unique identification number was provided to each participant and stored together with the survey results, in order to eliminate duplicate entries. The participants were given the option to save their responses and return to complete the survey, or they could edit or clear the replies and initiate the survey another time. All no respondents received email reminders. The response rate is estimated to be 31%. Data was recorded in the system using a password-protected data extraction form.

Variables and Measurement

Guided by Wilson's model of information-seeking behavior [38], the previous survey on services for supporting family carers of older dependent people in Europe "EUROFAMCARE" [39], and empirical evidence in the literature [16-28], this study included the following sets of independent variables: caregiver's demographics; caregiver's socioeconomic resources; and caregiving context. The dependent variable in this study is informal caregivers' frequent use of the Internet to access digital support services. In the survey, caregivers were asked to report how

frequently they were using the Internet to access digital support services. Those using the Internet at least several times per month to access digital support services were classified as “frequent users”, while those accessing it less often were classified as “infrequent users”. Three demographic measures were included: caregiver’s age, caregiver’s gender and caregiver’s health status. Ages were measured in chronological years and grouped into three categories: 18 to 39, 40 to 59, and 60 or older. Gender was measured nominally and was grouped into male and female. Caregiver’s health status was grouped into poor, fair and good. Measures of social and economic circumstances were the caregiver's educational attainment and their total household income. Educational attainment was grouped into primary, secondary, bachelor's degree and higher than bachelor's degree. Income was assessed by asking the caregiver about their “monthly household net income from all sources”. In order to distribute the income by different income groups and enhance the cross-national comparability of results between the two countries involved in this study, Italy and Sweden, we referred to the official figures of the national median equivalized disposable annual income from the European Commission’s European statistical system “Eurostat” [32]. We used these official figures in classifying the participants into three groups of household net income in each of these two countries:

- lower income group: income is less than below 50% of the national median equivalized disposable annual income. This is equivalent to an income lower than 5802 Euro in the case of Italy and an income lower than 9356 Euro in the case of Sweden;
- middle income group: income is between below 50 % of the national median equivalized disposable annual income and above 60 % of the national median equivalized disposable annual income. This is equivalent to an income between 5802 Euro and 19658 Euro in the case of Italy and an income between 9356 Euro and 26826 Euro in the case of Sweden;
- upper income group: income is higher than 19658 Euro in the case of Italy and higher than 26826 Euro in Sweden.

Caregiving context was assessed using the following variables: reported number of weekly hours of care provided to the care recipient; reported number of years spent providing care; age and gender of the care recipient; relationship between the care recipient and the caregiver; and the level of dependency of the care recipient. Responses concerning the average number of weekly hours of caregiving have been grouped into four categories: 1) 10 hours or less, 2) 11 to 20 hours, 3) 21 to 40 hours and 4) more than 40 hours. Care duration was measured on the basis of the caregiver’s reported length of care provision to the care recipient (in number of years), and respondents were classified into two groups: those caring for two years or less; and those caring for a longer time. The age of the care recipient was reported according to two groups: 60 years or less and more than 60 years. The gender of care recipients was grouped into male and female. Caregivers were requested to provide information about the person whom they care for, in order to

For peer review only

assess the relationship with the care recipient (e.g. parents / parents-in-law, spouse/partner, friend/neighbor, child or other relative. The level of dependency of the care recipient on the caregiver was clustered in 2 groups: high dependency and low dependency.

Data Analysis

The data analysis was conducted in three stages. It began with univariate analyses including percentages to describe the characteristics of this sample of caregivers. At the second stage, the relationship between the outcome variable and the independent variables was examined using Pearson's chi-squared test with Yates' continuity correction. Differences between groups were considered significant at the 5% level ($p \leq 0.05$). Contingency tables have been assessed, before proceeding to logistic regression, to ensure there were no cells with expected frequencies of fewer than 5 to prevent biased estimates [40]. At the last stage, logistic regression analysis was used to establish the ability of each variable to predict caregivers' frequent use of the Internet to access digital support services while controlling the effects of other variables. Variables identified as statistically significant in the bivariate analysis were entered into logistic regression analysis for each measure of use of the Internet to access digital support services.

The logistic regression analyses produced odds ratios with 95% confidence intervals to identify predictors of each measure. Results are reported in odds ratios, which can be interpreted as the ratio of the probability that caregivers with a particular characteristic (e.g., male gender) will use the Internet frequently to access digital support services, over the probability they will use the Internet frequently to access digital support services, had they not this characteristic. Odds ratios that are higher than 1 indicate a positive association between a given variable and using the Internet frequently to access digital support services, while an odds ratio lower than 1 indicates a negative association. Statistical analyses were performed using SPSS software version 28.0 (IBM, Armonk, NY, USA).

Research Ethics Approval

Permission to conduct the study was granted by the ethics committee of the faculty of economics, Marche Polytechnic University and was approved by the executive board on November 2, 2020 (1026353). Informal caregivers expressing interest in participating in the study were informed about the aim of the study, the expected time to complete the questionnaire, and that data would be stored by the Centre for Socio-Economic Research on Ageing of the Italian National Institute of Health and Science on Ageing. The technical functionality of the online questionnaire had been tested before fielding the questionnaire. The estimate time for survey completion was 10-15 minutes. Informed consent was obtained from all participants. No personal information about the participants such as their name or their IP address were collected. All the responses were anonymous.

For peer review only - http://bmjopen.bmj.com/site/about/guidelines.xhtml

Patient and public involvement

Patients or the public were not involved in the design, or conduct, or reporting, or dissemination plans of our research.

Results

Sample Description

A total of 663 informal caregivers, 410 from Italy and 253 from Sweden, participated in the survey by completing the online questionnaire. Table 1 presents the overall characteristics of the sample. Females represented a majority of respondents in the Italian group. The median age of caregivers was 54 years while the median age of care recipients was 73 years. Most Italian participants were providing care to a parent (n = 163, 39.8%), to a female care recipient (n=223, 54.4%), spent more than 40 hours per week providing care (n=170, 41.5%) and had completed secondary school or lower (n = 254, 62%). Nearly half of the participants (n=196, 47.8%) had an annual household income of less than 19.658 Euro. The big majority of caregivers in the Italian sample (n=342, 83.4%) reported a fair or poor health status, provided care to a highly-dependent care recipient (n=329, 80.2%) and had been providing care for more than 2 years (n=287, 70%) (Table 1).
When compared to their Italian counterparts, both Swedish participants and their care recipients had a higher median age of 65 and 75 years respectively. Females made up a majority of participants in the Swedish sample. Most of the Swedish respondents reported providing care to a spouse/partner (n=97, 38.3%), a male care recipient (n=136, 53.8%), spent less than 10 hours per week providing care (n=112, 44.3%) and had completed a secondary school or lower (n = 149, 58.9%). Nearly half of the participants in the Swedish group (n=109, 43.1%) had annual household incomes less than 26.826 Euro. The majority of the caregivers in the

For peer review only

Swedish sample (n=210, 83%) had a fair or poor health status, were caring for a highly dependent care recipient (n=139, 54.9%) and had been providing care for more than 2 years (n=142, 56.1%) (Table 1).

Table 1. Characteristics of the sample (total sample N =663)

**Variables**	**Italian sample n = 410 n (%)**	**Swedish sample n = 253 n (%)**
**Gender**		
Male	93 (22.7)	57 (22.5)
Female	317 (77.3)	196 (77.5)
**Age**		
Median	54	65
18-39	48 (11.7)	17 (6.7)
40-59	241 (58.8)	77 (30.4)
More than 60	121 (29.5)	159 (62.8)
**Health Status**		
Good	68 (16.6)	43 (17.0)
Fair	171 (41.7)	155 (61.3)
Poor	171 (41.7)	55 (21.7)
**Education**		
Primary	29 (7.1)	35 (13.8)
Secondary	225 (54.9)	114 (45.1)
Bachelor	114 (27.8)	61 (24.1)
Higher than bachelor's degree	42 (10.2)	43 (17.0)
**Income**		
Lower	39 (9.5)	17 (6.7)
Middle	157 (38.3)	92 (36.4)
Upper	214 (52.2)	144 (56.9)
**Care recipient relationship to caregiver**		
Parents (In law)	163 (39.8)	63 (24.9)
Spouse/Partner	64 (15.6)	97 (38.3)
Child	105 (25.6)	48 (19.0)
Friend/Neighbor	30 (7.3)	26 (10.3)
Other	48 (11.7)	19 (7.5)
**Gender of care recipient**		
Male	187 (45.6)	136 (53.8)
Female	223 (54.4)	117 (46.2)

For peer review only

**Age of care recipient**		
Median	73	75
60 or younger	160 (39.0)	73 (28.9)
More than 60	250 (61.0)	180 (71.1)
**Level of dependency of the care recipient**		
High dependency	329 (80.2)	139 (54.9)
Low dependency	81 (19.8)	114 (45.1)
**Hours spend caring each week**		
10 hours or less	115 (28.0)	112 (44.3)
11-20 hours	68 (16.6)	62 (24.5)
21-40 hours	57 (13.9)	30 (11.9)
More than 40 hours	170 (41.5)	49 (19.4)
**Number of years providing care**		
2 years or less	123 (30.0)	111 (43.9)
More than 2 years	287 (70.0)	142 (56.1)

Factors associated with caregivers' frequent use of the Internet to access digital support services

Table 2 shows the factors associated with caregivers' frequent use of the Internet to access digital support services in the bivariate analysis for each of the two countries of the study. In the Italian group, two-thirds of the respondents reported using the Internet at least several times per month to access digital support services. At the bivariate level, this was associated with two demographic variables, caregiver's age and health status, and two socio-economic measures, caregiver's educational attainment and total household income. Five measures of caregiving context – care recipient relationship to the caregiver, gender of care recipient, age of care recipient, hours per week spent caring and the level of dependency of the care recipient – were also linked to the frequent use of the Internet to access digital support services.

In the Swedish sample, 54.2 % of the participants reported using the Internet at least several times per month to access digital support services. In the bivariate analysis, caregiver's age was significantly associated with the frequent use of the Internet to access digital support services. Three measures of caregiving context were also linked with the frequent use of the Internet to access digital support services: care recipient relationship to the caregiver, age of care recipient and the number of hours spent caring each week. None of the measures of socio-economic

For peer review only

resources was significantly associated with frequent use of the Internet to access digital support services in the Swedish sample.

Table 2. Factors associated with caregivers' frequent use of the Internet to access digital support services in the bivariate analysis

	Using the Internet at least several times per month to access digital support services			
	Italian sample n = 410		Swedish sample n = 253	
**Variables**	**n (%)**	**p[1]**	**n (%)**	**p**
**All respondents**	274 (66.8)		137 (54.2)	
**Gender**		0.123		0.344
Male	56 (60.2) [2]		34 (59.6)	
Female	218 (68.8)		103 (52.6)	
**Age**		0.010		0.035
18-39	23 (47.9)		6 (35.3)	
40-59	169 (70.1)		50 (64.9)	
More than 60	82 (67.8)		81 (50.9)	

For peer review only

**Health Status**		0.042		0.268
Good	37 (54.4)		35 (63.6)	
Fair	115 (67.3)		79 (51.0)	
Poor	122 (71.3)		23 (53.5)	
**Education**		0.008		0.901
Primary	12 (41.4)		20 (57.1)	
Secondary	161 (71.6)		60 (52.6)	
Bachelor's degree	76 (66.7)		32 (52.5)	
Higher than bachelor's degree	25 (59.5)		25 (58.1)	
**Income**		0.025		0.736
Lower	32 (82.1)		10 (58.8)	
Middle	110 (70.1)		47 (51.1)	
Upper	132 (61.7)		80 (55.6)	
**Care recipient relationship to caregiver**		< 0.001		0.014
Parents (In law)	95 (58.3)		23 (36.5)	
Spouse/Partner	49 (76.6)		55 (56.7)	
Child	88 (83.8)		33 (68.8)	
Friend/Neighbor	16 (53.3)		15 (57.7)	
Other	26 (54.2)		11 (57.9)	
**Gender of care recipient**		< 0.001		0.732
Male	141 (75.4)		75 (55.1)	
Female	133 (59.6)		62 (53.0)	
**Age of care recipient**		0.002		0.037
60 or younger	121 (75.6)		47 (64.4)	
More than 60	153 (61.2)		90 (50.0)	

For peer review only

**Level of dependency of the care recipient**<br>High dependency<br>Low dependency	228 (69.3)<br>46 (56.8)	0.032	76 (54.7)<br>61 (53.5)	0.853
**Hours spend caring each week**<br>10 hours or less<br>11-20 hours<br>21-40 hours<br>More than 40 hours	57 (49.6)<br>46 (67.6)<br>38 (66.7)<br>133 (78.2)	< 0.001	49 (43.8)<br>38 (61.3)<br>17 (56.7)<br>33 (67.3)	0.022
**Number of years providing care**<br>2 years or less<br>More than 2 years	79 (64.2)<br>195 (67.9)	0.464	65 (58.6)<br>72 (50.7)	0.213

Notes:
[1] Differences between groups were considered significant at the 5% level ($p \leq 0.05$)
[2] Male caregivers who are frequently using the Internet as a % of the total number of male caregivers in the sample.

Predictors of caregivers' frequent use of the Internet to access digital support services

Table 3 summarizes the results of the logistic regression analysis predicting caregivers' frequent use of the Internet to access digital support services. For the Italian sample, nine variables significantly associated with a frequent use of the Internet to access digital support services in the bivariate analysis were entered into logistic regression analysis to identify which were predictive: caregiver's age, health status, educational attainment, total household income, care recipient relationship to the caregiver, gender of care recipient, age of care recipient, number of weekly hours of care and the level of dependency of the care recipient. The multivariate analysis indicated that educational attainment, number of weekly hours of care, total household income, care recipient relationship to the caregiver and gender of care recipient remained significant predictors. The strongest predictor was the educational attainment of the caregivers. Informal caregivers who completed education equivalent to a Bachelor's degree level had 3.649 times the odds of using the Internet at least several times per month to access digital support services compared to those who completed a primary education (p=0.007, 95%CI: 1.424-9.350). Caregivers who spend more than 40 hours per week providing care were almost 3 times more likely to be frequent users of the Internet to access digital support services in comparison with those who spend 10 hours or less per week providing care. The odds of frequent use of the Internet to access digital support

services were 2.646 times higher for caregivers belonging to the lower household income group compared to caregivers belonging to the upper household income group (p=0.040, 95%CI: 0.149-0.957). Regarding the relationship between the caregiver and care recipient, the caregivers of a child had 2.895 times the odds of using the Internet at least several times per month to access digital support services compared to those who provide care to another relative (p = 0.042, 95% CI: 1.037-8.083). The odds of frequently accessing digital support services were 1.739 times higher for caregivers who provide care to a male care recipient compared to those providing care to a female care recipient (p = 0.023, 95% CI: 0.356-0.928).

The logistic regression analysis to predict the frequent use of the Internet to access digital support services among Swedish participants consisted of the four statistically significant factors identified in the bivariate analysis: caregiver's age, care recipient relationship to the caregiver, age of care recipient and the number of weekly hours of care (Table 3). The number of weekly hours of care remained a significant predictor in the multivariate analysis for the Swedish sample and was the strongest predictor. Swedish respondents who spend more than 40 hours per week providing care were almost 2.5 times more likely to be frequent users of the Internet to access digital support services as opposed to those who dedicate 10 hours or less per week to care provision (p = 0.027, 95% CI: 1.105-5.218). The age of the caregiver also remained a significant predictor in the multivariate analysis. Caregivers in the age group 40-59 years were 2.237 times more likely to use the Internet at least several times per month to access digital support services in comparison with those of the age group 60+ years (p = 0.018, 95% CI: 1.150-4.352).

Table 3. Multivariate logistic regressions: caregivers' frequent use of the Internet to access digital support services

	**Using the Internet at least several times per month to access digital support services**					
	**Italian sample n = 410**			**Swedish sample n = 253**		
**Variables**	**p Value**	**OR**	**95% CIs**	**p Value**	**OR**	**95% CIs**
**Age (in years) (Ref.: 60+)**						
18-39	0.270	0.630	0.277-1.433	0.653	0.761	0.231-2.508
40-59	0.563	1.175	0.680-2.030	0.018	2.237	1.150-4.352

For peer review only

**Health Status (Ref.: Good)**				- [1]	-	-
Fair	0.703	1.105	0.661-1.850			
Poor	0.925	1.033	0.523-2.040			
**Education (Ref.: Primary)**				-	-	-
Secondary	0.008	3.236	1.358-7.711			
Bachelor	0.007	3.649	1.424-9.350			
Higher than bachelor's degree	0.077	2.624	0.901-7.647			
**Income (Ref.: Lower)**				-	-	-
Middle	0.170	0.514	0.198-1.331			
Upper	0.040	0.378	0.149-0.957			
**Care recipient relationship to caregiver (Ref.: Other)**						
Parents (In law)	0.554	0.797	0.376-1.688	0.086	0.370	0.119-1.150
Spouse/Partner	0.337	1.611	0.608-4.267	0.634	0.777	0.275-2.196
Child	0.042	2.895	1.037-8.083	0.911	1.075	0.302-3.828
Friend/Neighbor	0.673	0.806	0.297-2.192	0.885	1.095	0.320-3.744
**Gender of care recipient (Ref.: Male)**				-	-	-
Female	0.023	0.575	0.356-0.928			

**Age of care recipient (Ref.: 60 or younger)**						
More than 60	0.211	1.616	0.762-3.424	0.920	1.046	0.436-2.511
**Level of dependency of the care recipient (Ref.: High dependency)**				-	-	-
Low dependency	0.738	1.111	0.599-2.062			
**Hours spend caring each week (Ref.: 10 hours or less)**						
11-20 hours	0.021	2.241	1.127-4.459	0.085	1.822	0.921-3.602
21-40 hours	0.103	1.908	0.878-4.144	0.311	1.568	0.656-3.748
More than 40 hours	0.002	2.928	1.481-5.791	0.027	2.401	1.105-5.218

Notes:

Only variables significantly associated with using the Internet frequently to access digital support services in the bivariate analysis were entered into multivariate logistic regression analysis

Discussion

Principal findings

The purpose of this study was to identify important factors related to caregivers' use of the Internet to access digital support services in Italy and Sweden. The findings suggest that a number of demographic, socio-economic and caring circumstances are associated with the frequency of using the Internet to access digital support services among caregivers in both countries. Multivariate regression analyses enabled the effect of confounding factors to be controlled for and predictors of use to be identified. In consistency with literature on the same topic in different countries [16-28], our findings indicate that caregiver's age, health status, caregiver's educational attainment, total household income, care recipient relationship to the caregiver, gender of care recipient, age of care recipient, hours per week spent caring and the level of dependency of the care recipient are all associated with use.

The study shows that more than half of the caregivers in both countries frequently use the Internet to access digital support services. While the use of the Internet for health information has been somewhat less common in Southern European

For peer review only - http://bmjopen.bmj.com/site/about/guidelines.xhtml

countries, in our study the Italian and the Swedish groups report a similar use of the Internet to access digital support services. This may be related to the lower median age of the Italian sample compared to the Swedish one. Caregivers from Southern European countries with a family-based care system often lack support in terms of formal services and professional training from the government [41-48]. This shortcoming of support may increase their need for information and services. Digital support services may be an alternative support source that enables remote access to information and training about care and caring-related issues. Previous studies suggested that the use of the Internet for health information in Southern European countries is increasing, and that caregivers from this region are showing an increased interest in accessing new technologies aiming to support them [48-50]. In both countries, most of the caregivers who participated in the study were females, which is consistent with the results of previous works [25-28] and with the central role played by females in the provision of informal care [2-4]. In coherence with previous literature [39,41-48], the majority of Italian participants in our study provided care to a parent (in law) and spent more than 40 weekly hours of care, compared to their Swedish counterparts who provided care to spouse/partner and spent less than 10 hours per week providing care. Previous research showed that care for someone in one's own household is more common in Southern European countries than in Northern countries. In Southern countries, caregivers are more likely to live with their care recipients who often are parents / in-laws [39,41-48]. In Northern countries, in-household care is mostly spouse care, as it is rare for old persons to live with anyone else than their spouse. Consequently, caregivers from Southern European countries spend more hours in caregiving compared to caregivers from Northern countries.

As it is to be expected given previous research on using the Internet for general health information [19-24], the digital divide may negatively affect caregivers' use of the Internet to access digital support services [51,52]. The socioeconomic status of users seems to be a significant factor that increases the digital divide in Southern European countries [53-55]. This was apparent in our study, showing that the divide was more significant in the case of the Italian group compared to the Swedish one. While none of the measures of socio-economic resources was significantly associated with a frequent use of the Internet to access digital support services in the Swedish group, the strongest predictor for the frequency of Internet use in the Italian group was the caregiver's educational attainment. Previous research has shown that better-educated caregivers are more likely to be engaged in more frequent online activities [25-28]. Income was also a predictor for the frequency of Internet use to access digital support services in the Italian group, with higher odds for caregivers belonging to the lower household income group. While literature suggests that general Internet users in higher-income households are more likely than others to go online frequently [56,57], previous studies on the Internet use for health-related activities suggest that lower-income households may be more likely than others to go online for support activities [16,58,59]. One possible explanation is that those with higher incomes may have other means of support, while those with lower incomes may turn to the Internet as an alternative source of assistance.

For peer review only

The literature shows that age is a factor associated with Internet use [16-28]. In the Swedish group of our study, age is a significant predictor of frequency of use. Age remained an important predictor of use when the effects of other demographics, socio-economic factors and caring circumstances had been controlled for. This suggests that the relationship between age and use among Swedish caregivers cannot be entirely explained by increased financial hardship in later life. Previous research suggests that use of the Internet for health information is relatively constant by age, until age 65 when it begins to decline [16].

Patterns of use among caregivers in both countries also seem to be shaped by the caring experience. The number of weekly hours of care was a significant predictor for the frequency of Internet use by participants in both countries. Evidence from literature suggests that high-intensity caregivers report higher levels of information and service needs [60]. Given the availability and convenience of online sources, high-intensity caregivers may turn to the Internet for digital support services.

Limitations

Some limitations concerning this study need to be considered. The risk of the typical sampling bias should be mentioned as higher income and more educated caregivers are more likely to participate in research studies involving modern technologies, which was the case in our study. Furthermore, the sample size, especially of the Swedish sample, prevented us from carrying out more sophisticated statistical analyses. Moreover, not all of those who provide informal caregiving and assistance to others identify themselves as informal caregivers; consequently, we may have failed to capture the experiences of these underrepresented groups. We may have also failed to capture the concerns of the caregivers that may limit their ability in using digital resources. Although the most important variables identified from empirical evidence in the literature were included in the models, residual external variables may still have influenced our results. Conclusions drawn from this study results must be tempered by the fact that respondents were already possessing minimal digital skills that would enable them to access online services. It is possible that those who are not interested or involved with technology or those with limited digital access are less likely to respond to online surveys; consequently, the data collected online might be skewed and the sample might be less representative for the population. These issues might have influenced our findings and underline the need to interpret the findings from this study and other studies on caregivers with some caution when generalizing the findings.

Recommendations and Implications

Our results indicate that digital support services may enable remote access to information and training about care and caring-related issues. In this context, looking for information and support services online may be considered an attempt to close some knowledge gap. With the rapid technology advancement and increased access to the Internet, more caregivers are expected to access these services [61]. This suggests that the interaction with informal caregiver by health

For peer review only

care professionals and other parties with an interest in supporting them (e.g. caregiver advocacy organizations) is an integral part of the value chain that supports both communication and coordination of services. Hence, these parties should all be more engaged with developing digital support services targeted at informal caregivers, and carefully assess and identify their information and service needs. Consequently, better targeted information could be provided to caregivers through credible online sources. In this regard, an early assessment of caregivers' needs and digital skills demonstrates that large-scale actions aiming to equip informal caregivers with the digital skills they need to access digital support services are needed. This is key to enable informal caregivers to identify the available digital support services, and apply them to their own care situation. More research is therefore needed to examine the extent to which existing digital support services meet caregivers' information and service needs.

Addressing socio-economic inequalities is likely to be key to reduce the digital divide in caregivers' use of the Internet to access digital support services. As for the influence of age and education on the digital divide, health care professionals, service providers and social workers should pay particular attention to those caregivers who are older and less educated. Access to computers and Internet connections at public facilities, such as local libraries, community centers and senior centers should be provided with extra support to accommodate caregivers' information needs and overcome any barriers of use [15,19]. Moreover, seminars and campaigns on how to access digital support services could enhance caregivers' digital skills and experiences. Tailor-made campaigns and classes for older and less-educated adults are needed to help address any barriers related to their use of computers and new technology.

Poor connectivity to the internet, particularly for informal caregivers in rural areas, is an obstacle to the use of any support service delivered over the internet. Policy makers should allocate funding for improving digital infrastructures in order to facilitate the deployment of digital support services and improve informal caregivers' access to these services. In this regard, an identification of sustainable business models, exchange of good practices, collection of evidence, and a transferability of optimal solutions among localities, regions, and countries are all important to continue allocating public funding for initiatives. Moreover, informal caregivers have concerns on data ownership and privacy of the data. Privacy concerns may be especially relevant to older informal caregivers, who voice the most concerns over the privacy and security of their information online. Digital support services should be sensitive to informal caregiver privacy concerns and the extent to which a technology might undermine their autonomy, control and dignity. In this context, blending online support with involving health care professionals in the provision of professional support leads to overcoming possibleskepticism.

The finding that caregivers who indicate higher-intensity levels of caregiving are likely to engage in frequent Internet use to access digital support services may suggest that the Internet could be used to reach out to these caregivers and meet their information and service needs. Online training materials, support groups, social networking systems for peer support and volunteer call networks could be used to reach out to caregivers [62]. Research is needed to further examine the

For peer review only

effectiveness of digital support services in helping caregivers, if we are to improve these services and tailor them to the lives of those with substantial and unpredictable caring responsibilities.

Conclusions

The findings from this study can provide guidance and assistance for the deployment of digital support services for informal caregivers. Nevertheless, due to rapid technological innovation, especially in this sector, continuous research needs to be conducted and guidelines for developing digital support services should be made adaptable to ongoing and future changes. The care sector is undergoing a fast transformation and expansion also due to the direct and indirect effects of the COVID-19 pandemic. Health and social care delivery systems experience a technologically supported transition towards home care. New technologies are being developed for informal caregivers and these tools may well offer benefits to many of them. It is widely acknowledged that caregivers are a group with high levels of unmet needs when it comes to their access to information and other services. Digital support services could be important tools to empower and support informal caregivers. On the other hand, it also needs to be recognized that informal caregivers are a diverse population, living in a wide range of personal and social circumstances. When it comes to policy and practice in relation to caregivers, similarly to other broad vulnerable groups, there is no 'one size fits all' approach, and it is therefore important to consider the specific characteristics and needs of both caregivers and care recipients. Policy makers, health care professionals and all parties with an interest in supporting informal caregivers are encouraged to identify the outcomes that the latter regard as helpful, and to identify the interventions that can achieve such outcomes in consultation with them. This applies as much to the approach taken in relation to the development of digital support services as it does to other services. While digital support services have the potential to meet some of the needs of the caregivers, they cannot be seen as the only way to deliver information and support. These services represent only one of many instrument in a toolbox, and should therefore be tailored in a coordinated way with other existing services, such as respite care, access to training, and recognition of skills and work-life balance measures.

Contributorship statement

AH developed the research idea and wrote the manuscript. GL and MH were involved in the conceptualization of the project and provided critical evaluation and approval of the final submitted manuscript

Competing interests

None declared

Funding and grant number

For peer review only

This research was funded by the European Union's Horizon 2020 research and innovation program under the Marie Skłodowska-Curie grant agreement number 814072 for the 4-year innovative training network ENTWINE informal care.

Data sharing statement

Raw data cannot be made openly available as the participants provided an informed consent, which indicated that the data to be collected shall not be disclosed.

References

1- Roth DL, Fredman L, Haley WE. Informal caregiving and its impact on health: A reappraisal from population-based studies. The Gerontologist. 2015.

2- Ciccarelli N, Van Soest A. Informal caregiving, employment status and work hours of the 50+ population in Europe. De Economist. 2018.

3- Colombo, F, Llena-Nozal, A, Mercier, J and Tjadens, F, 2011, Help Wanted? Providing and paying for long-term care, OECD Health Policy Studies, OECD Publishing.

4-Eurocarers. Enabling carers to care: an EU strategy to support and empower informal carers. 2020. URL: https://eurocarers.org/download/5315/Eurocarers-Strategy_final.pdf

5- Zigante, Valentina. 2018. 'Informal Care in Europe: Exploring Formalisation, Availability and Quality.' Brussels: LSE Consulting for the European Commission, 2018.

6- Lamura G, Mnich E, Nolan M, Wojszel B, Krevers B, Mestheneos L, EUROFAMCARE Group. Family carers' experiences using support services in Europe: empirical evidence from the EUROFAMCARE study. Gerontologist 2008.

7-Lamura G, Di Rosa M, Papa R, Poli A, Barbabella F. Implications of the use of migrant care work and web-based services on family caregivers' health. Int J Care Caring 2019 Feb 28.

8-Carretero S, Stewart J, Centeno C, Barbabella F, Schmidt A, Lamontagne-Godwin F, et al. "Can technology-based services support long-term care challenges in home care?" Analysis of evidence from social innovation good practices across the EU: CARICT project summary report. 2012

9-Barbabella F, Schmidt A, Lamontagne-Godwin F, Rodrigues R, Ruppe G, Lamura G. Assessing the impact of ICT-based solutions for carers in europe: preliminary findings from the CARICT project.

10- Kluzer S, Redecker C, Mediavilla C. Long-term care challenges in an ageing society: The role of ICT and migrants results from a study on England, Germany, Italy and Spain (No. JRC58533). 2010.

11-Newman K, Wang AH, Wang AZ, Hanna D. The role of internet-based digital tools in reducing social isolation and addressing support needs among informal caregivers: a scoping review. BMC public health. 2019 Dec;19.

12-Carretero S, Stewart J, Centeno C. Information and communication technologies for informal carers and paid assistants: benefits from micro-, meso-, and macro-levels. Eur J Ageing 2015.

13-Read J, Blackburn C. Carers' perspectives on the internet: implications for social and health care service provision. British Journal of Social Work. 2005.

14- Washington, K., Meadows, S., Elliott, S., & Koopman, R. (2011). Information needs of informal caregivers of older adults with chronic health conditions. Patient Education and Counseling,
15- Hassan AY. Challenges and recommendations for the deployment of information and communication technology solutions for informal caregivers: scoping review. JMIR aging. 2020.
16- Bundorf MK, Wagner TH, Singer SJ, Baker LC. Who searches the internet for health information?. Health services research. 2006.
17- Basch EM, Thaler HT, Shi W, Yakren S, Schrag D. Use of information resources by patients with cancer and their companions. Cancer: Interdisciplinary International Journal of the American Cancer Society. 2004.
18-Shaffer KM, Chow PI, Cohn WF, Ingersoll KS, Ritterband LM. Informal caregivers' use of internet-based health resources: An analysis of the health information national trends survey. JMIR aging. 2018.
19-Brodie M, Flournoy RE, Altman DE, Blendon RJ, Benson JM, Rosenbaum MD. Health Information, The Internet, And The Digital Divide: Despite recent improvements, Americans' access to the Internet—and to the growing body of health information there—remains uneven. Health affairs. 2000.
20- Czaja SJ, Sharit J, Hernandez MA, Nair SN, Loewenstein D. Variability among older adults in Internet health information-seeking performance. Gerontechnology. 2010.
21- Baker L, Wagner TH, Singer S, Bundorf MK. Use of the Internet and e-mail for health care information: results from a national survey. Jama. 2003.
22-Ybarra M, Suman M. Reasons, assessments and actions taken: sex and age differences in uses of Internet health information. Health education research. 2008.
23- Bass SB, Ruzek SB, Gordon TF, Fleisher L, McKeown-Conn N, Moore D. Relationship of Internet health information use with patient behavior and self-efficacy: experiences of newly diagnosed cancer patients who contact the National Cancer Institute's Cancer Information Service. Journal of Health Communication. 2006.
24- Flynn KE, Smith MA, Freese J. When do older adults turn to the internet for health information? Findings from the Wisconsin Longitudinal Study. Journal of general internal medicine. 2006.
25- Li H. Informal caregivers' use of the internet for caregiving information. Social work in health care. 2015.
26- Meyer K, Gassoumis ZD, Kelly K, Benton D. What are the characteristics of caregivers logging in for support services?. Innovation in aging. 2019.
27- Buchanan RJ, Huang C, Crudden A. Use of the internet by informal caregivers assisting people with multiple sclerosis. Journal of technology in human services. 2012.
28- Blackburn C, Read J, Hughes N. Carers and the digital divide: factors affecting Internet use among carers in the UK. Health & Social Care in the Community. 2005.
29-Garcés J, Ródenas F, Hammar T. Converging methods to link social and health care systems and informal care—confronting Nordic and Mediterranean approaches. In Long-Term Care in Europe 2013.

For peer review only

30- Nies H, Leichsenring K, Mak S. The emerging identity of long-term care systems in Europe. InLong-Term Care in Europe 2013 . Palgrave Macmillan, London.
31- Schulmann K, Leichsenring K, Genta M, Grigaliūnienė Ž, Kucsera C, Matei A, Määttänen N, Naegele G, Paat G, Ahi SP, Principi A. Social support and long-term care in EU care regimes. Framework Conditions and Initiatives of Social Innovation in an Active Ageing Perspective. Overview Report. WP8 MoPAct Project. 2014.
32- European Commission's European statistical system "Eurostat": https://ec.europa.eu/eurostat/web/main/home
33-World Health Organization . Italy: WHO Statistical Profile. World Health Organization; Geneva, Switzerland: 2015.
34-World Health Organization . Sweden: WHO Statistical Profile. World Health Organization; Geneva, Switzerland: 2015.
35-Eurocarers. Country profiles: Italy.
https://eurocarers.org/country-profiles/italy/
36-Eurocarers. Country profiles: Sweden.
https://eurocarers.org/country-profiles/Swden/
37- Draguet V. EU4Health 2021–2027–a vision for a healthier European Union. Public Health–European Commission
38- Wilson TD. Models in information behaviour research. Journal of documentation. 1999 Aug 1.
39- Lamura G, Mnich E, Nolan M, Wojszel B, Krevers B, Mestheneos L, Döhner H. Family carers' experiences using support services in Europe: empirical evidence from the EUROFAMCARE study. The Gerontologist. 2008.
40- Haberman SJ. A warning on the use of chi-squared statistics with frequency tables with small expected cell counts. Journal of the American Statistical Association. 1988.
41-Ferre F, de Belvis AG, Valerio L, Longhi S, Lazzari A, Fattore G, et al. Italy: health system review. Health Syst Transit. 2014.
42- Sansoni J, Vellone E, Piras G. Anxiety and depression in community-dwelling, Italian Alzheimer's disease caregivers. Int J Nurs Pract. 2004.
43- Wulff J, Fänge AM, Lethin C, Chiatti C. Self-reported symptoms of depression and anxiety among informal caregivers of persons with dementia: a cross-sectional comparative study between Sweden and Italy. BMC Health Services Research. 2020.
44-Castiello M, del Barrio É, Castejon P, Tortosa M, Sundström G, Malmberg B, Johansson L. Family care for elders in Europe: Policies and practices.
45- Quattrini S, Melchiorre MG, Balducci C, Spazzafumo L, Lamura G. Services for Supporting Family Carers of Older Dependent People in Europe: Characteristics, Coverage and Usage. The National Survey Report for Italy. 2006.
46- Szinovacz ME, Davey A, editors. Caregiving contexts: Cultural, familial, and societal implications. Springer Publishing Company; 2007.
47- Glendinning C, Tjadens F, Arksey H, Morée M, Moran N, Nies H. Care provision within families and its socio-economic impact on care providers. Heslington, York: Social Policy Research Unit, University of York; 2009.
48- Eurocarers/IRCCS-INRCA (2021). Impact of the COVID-19 outbreak on informal carers across Europe – Final report. Brussels/Ancona.

For peer review only

49- Kummervold PE, Wynn R. Health information accessed on the internet: the development in 5 European countries. International Journal of Telemedicine and Applications. 2012.
50- Athanasopoulou C, Välimäki M, Koutra K, Löttyniemi E, Bertsias A, Basta M, Vgontzas AN, Lionis C. Internet use, eHealth literacy and attitudes toward computer/internet among people with schizophrenia spectrum disorders: a cross-sectional study in two distant European regions. BMC medical informatics and decision making. 2017.
51-Burton LC, Newsom JT, Schulz R, Hirsch CH, German PS. Preventive health behaviors among spousal caregivers. Preventive medicine. 1997.
52-Given B, Sherwood PR, Given CW. What knowledge and skills do caregivers need?. Journal of Social Work Education. 2008.
53- Gourova E, Antonova A. Bridging the digital divide in South-Eastern Europe. IV International Bulgarian-Greak Scientific Conference Computer Science. 2008.
54-Dutta S, Geiger T, Lanvin B. The global information technology report 2015. In World Economic Forum. 2015.
55- Polykalas SE. Assessing the evolution of the digital divide across European Union. In2014 International Conference on Web and Open Access to Learning (ICWOAL) 2014.
56- Bansil P, Keenan NL, Zlot AI, Gilliland JC. Peer reviewed: health-related information on the web: results from the HealthStyles Survey, 2002–2003. Preventing chronic disease. 2006.
57- Jansen J. Use of the internet in higher-income households. Washington, DC: Pew Research Center; 2010.
58- Atkinson N, Saperstein S, Pleis J. Using the internet for health-related activities: findings from a national probability sample. Journal of medical Internet research. 2009.
59-Lee SY, Hwang H, Hawkins R, Pingree S. Interplay of negative emotion and health self-efficacy on the use of health information and its outcomes. Communication Research. 2008.
60- Li H. Unmet service needs: a comparison between dementia and non-dementia caregivers. Home health care services quarterly. 2012.
61-Fox S, Brenner J. Family caregivers online. Washington, DC: Pew Internet & American Life Project. 2012.
62- White MH, Dorman SM. Online support for caregivers. Analysis of an Internet Alzheimer mail group. Computers in nursing. 2000.

For peer review only

CHERRIES Checklist

Item Category	Checklist item	Page no.
**Target Population**	Study Design	5-7
**Ethics**	Study Design	8
	Consent Form	8
	Data Protection	8
**Development and pre-testing**	Data collection	5-8
**Recruitment Process and survey administration**	Data Collection	5-8

For peer review only

**Responses rate**	Results	8
**Preventing multiple entries from the same individual**	Data collection	6
**Analysis**	Statistical analysis and data handling	6-8

Notes: Adapted from: Eysenbach G. Improving the quality of Web surveys: the Checklist for Reporting Results of Internet E-Surveys (CHERRIES). *J Med Internet Res*. 2004;6(3):e34. doi: 10.2196/jmir.6.3.e34. PMID: 15471760. PMCID: PMC1550605. Available from: https://www.jmir.org/2004/3/e34/. ©Gunther Eysenbach. Originally published in the Journal of Medical Internet Research (http://www.jmir.org), 29.9.2004. Creative Commons Attribution License (http://www.creativecommons.org/licenses/by/2.0/).

For peer review only

For peer review only

STROBE Statement—Checklist of items that should be included in reports of ***cross-sectional studies***

	Item No	Recommendation	Page No
**Title and abstract**	1	(*a*) Indicate the study's design with a commonly used term in the title or the abstract	1
		(*b*) Provide in the abstract an informative and balanced summary of what was done and what was found	1-2
**Introduction**			
Background/rationale	2	Explain the scientific background and rationale for the investigation being reported	3-4
Objectives	3	State specific objectives, including any prespecified hypotheses	3-4
**Methods**			
Study design	4	Present key elements of study design early in the paper	5
Setting	5	Describe the setting, locations, and relevant dates, including periods of recruitment, exposure, follow-up, and data collection	5
Participants	6	(*a*) Give the eligibility criteria, and the sources and methods of selection of participants	5
Variables	7	Clearly define all outcomes, exposures, predictors, potential confounders, and effect modifiers. Give diagnostic criteria, if applicable	6
Data sources/ measurement	8*	For each variable of interest, give sources of data and details of methods of assessment (measurement). Describe comparability of assessment methods if there is more than one group	*6*
Bias	9	Describe any efforts to address potential sources of bias	6
Study size	10	Explain how the study size was arrived at	6
Quantitative variables	11	Explain how quantitative variables were handled in the analyses. If applicable, describe which groupings were chosen and why	6-7
Statistical methods	12	(*a*) Describe all statistical methods, including those used to control for confounding	7-8
		(*b*) Describe any methods used to examine subgroups and interactions	7-8
		(*c*) Explain how missing data were addressed	7-8
		(*d*) If applicable, describe analytical methods taking account of sampling strategy	7-8
		(*e*) Describe any sensitivity analyses	7-8
**Results**			
Participants	13*	(a) Report numbers of individuals at each stage of study—eg numbers potentially eligible, examined for eligibility, confirmed eligible, included in the study, completing follow-up, and analysed	9
		(b) Give reasons for non-participation at each stage	9
		(c) Consider use of a flow diagram	9
Descriptive data	14*	(a) Give characteristics of study participants (eg demographic, clinical, social) and information on exposures and potential confounders	10-11
		(b) Indicate number of participants with missing data for each variable of interest	10-11
Outcome data	15*	Report numbers of outcome events or summary measures	12-17

For peer review only

Main results	16	(*a*) Give unadjusted estimates and, if applicable, confounder-adjusted estimates and their precision (eg, 95% confidence interval). Make clear which confounders were adjusted for and why they were included	12-17
		(*b*) Report category boundaries when continuous variables were categorized	12-17
		(*c*) If relevant, consider translating estimates of relative risk into absolute risk for a meaningful time period	12-17
Other analyses	17	Report other analyses done—eg analyses of subgroups and interactions, and sensitivity analyses	12-17
**Discussion**			
Key results	18	Summarise key results with reference to study objectives	18-19
Limitations	19	Discuss limitations of the study, taking into account sources of potential bias or imprecision. Discuss both direction and magnitude of any potential bias	19-20
Interpretation	20	Give a cautious overall interpretation of results considering objectives, limitations, multiplicity of analyses, results from similar studies, and other relevant evidence	20-21
Generalisability	21	Discuss the generalisability (external validity) of the study results	20-21
**Other information**			
Funding	22	Give the source of funding and the role of the funders for the present study and, if applicable, for the original study on which the present article is based	22

*Give information separately for exposed and unexposed groups.

Note: An Explanation and Elaboration article discusses each checklist item and gives methodological background and published examples of transparent reporting. The STROBE checklist is best used in conjunction with this article (freely available on the Web sites of PLoS Medicine at http://www.plosmedicine.org/, Annals of Internal Medicine at http://www.annals.org/, and Epidemiology at http://www.epidem.com/). Information on the STROBE Initiative is available at www.strobe-statement.org.

For peer review only
